# Generative models for protein sequence modeling: recent advances and future directions

**DOI:** 10.1093/bib/bbad358

**Published:** 2023-10-20

**Authors:** Mehrsa Mardikoraem, Zirui Wang, Nathaniel Pascual, Daniel Woldring

**Affiliations:** Michigan State University (MSU)‘s Department of Chemical Engineering and Materials Science; Regeneron Pharmaceuticals, Inc. Having received his B.S. in Chemical Engineering from MSU, he is currently pursuing a M.S. in Computer Science from Syracuse University; B.S. in Chemical Engineering from MSU; MSU’s Department of Chemical Engineering and Materials Science and a member of MSU’s Institute for Quantitative Health Sciences and Engineering

**Keywords:** generative machine learning (ML) models, protein engineering, generative adversarial neural networks (GANs), variational autoencoders (VAE), natural language processing (NLP), diffusion models

## Abstract

The widespread adoption of high-throughput omics technologies has exponentially increased the amount of protein sequence data involved in many salient disease pathways and their respective therapeutics and diagnostics. Despite the availability of large-scale sequence data, the lack of experimental fitness annotations underpins the need for self-supervised and unsupervised machine learning (ML) methods. These techniques leverage the meaningful features encoded in abundant unlabeled sequences to accomplish complex protein engineering tasks. Proficiency in the rapidly evolving fields of protein engineering and generative AI is required to realize the full potential of ML models as a tool for protein fitness landscape navigation. Here, we support this work by (i) providing an overview of the architecture and mathematical details of the most successful ML models applicable to sequence data (e.g. variational autoencoders, autoregressive models, generative adversarial neural networks, and diffusion models), (ii) guiding how to effectively implement these models on protein sequence data to predict fitness or generate high-fitness sequences and (iii) highlighting several successful studies that implement these techniques in protein engineering (from paratope regions and subcellular localization prediction to high-fitness sequences and protein design rules generation). By providing a comprehensive survey of model details, novel architecture developments, comparisons of model applications, and current challenges, this study intends to provide structured guidance and robust framework for delivering a prospective outlook in the ML-driven protein engineering field.

## INTRODUCTION

Proteins are genetically encoded macromolecules that regulate biological systems. The diverse size and chemical composition of proteins enable diverse functionality. Therefore, effectively engineered proteins with modified functions may serve in various fields from cosmetics to environmental bioremediation. Engineered proteins can be optimized to target disease biomarkers for the early detection of cancer, Alzheimer’s and inflammatory diseases [1–5]. Protein-based therapeutics is also another significant application; namely serum therapy with therapeutic antibodies which started more than a century ago and evolved with scientific advancements. In addition, protein engineering is a practical tool to address environmental issues resulting from industrialization [6–9]. For example, heavy metal protein binders displayed on the bacterial surface are capable of remediating environments contaminated with heavy metals [7, 10]. Despite the promise of protein engineering to revolutionize medicine and industry, discovering proteins with desired functionalities is exceedingly challenging. Within the astronomical number of ways to build a protein (i.e. unique protein sequences), the vast majority lack function entirely. Moreover, making a random change to a functional protein is typically detrimental to its function and stability. This highlights a need for improved strategies in obtaining novel proteins with favorable properties such as high binding affinity and desired developability [11, 12]. While previous strategies such as energy-based scoring [13] and evolutionary [14] methods are still informative, they have drawbacks, including inaccurate modeling and search strategy inefficiency. Recent advancements in computational methods and the rapidly growing availability of protein sequence data facilitated the use of new, data-driven approaches for protein design and engineering [15, 16].

ML techniques may offer a promising route for navigating the high-dimensional landscape of protein design and engineering. They have shown a high success rate in various domains such as processing text, images and audio. In theory-driven approaches, the researcher obtains the domain knowledge of the problem that needs to be solved and produces mathematical models to capture the attributes and physics of the study. In contrast, ML methods are mainly centered on modeling the observed data while previous knowledge and theory may also be infused to the model. To assess how well a dataset is modeled via ML, a loss function is defined that measures the difference between the model’s prediction and real data. It then is optimized so that the prediction is close to reality (i.e. the difference between the model and actual data is minimized). Training a model involves fitting the model parameters to optimize the loss function. A trained ML model, therefore, is useful in understanding the given data and aiding in future decisions by identifying trends, predicting outcomes and recognizing anomalies. Machine learning models can be classified into two types of models—discriminative and generative. Discriminative models are ML models used to predict the conditional probability of labels based on the given data features. In contrast, generative models aim to discover how the data is constructed by estimating the joint probability distributions of features and corresponding labels. Deep learning (DL), a subset of ML that uses neural networks for the training, is particularly well-suited for complex domains since it can extract high-level features (i.e. features that cannot be interpreted by humans) from the given dataset [17].

Sparse high-fitness variants can be efficiently sampled from the vast, rugged protein fitness space landscape using DL techniques that implement statistical and probabilistic models [18]. For sequence-function mapping, protein sequences can be vectorized (e.g. with one-hot encodings or embeddings) to get proper input representations that are compatible with ML algorithms. Therefore, due to both high demand and compatibility, various ML models have been applied to predict protein binding affinity [19], thermostability [20], developability [12], solubility [21] and stability [22, 23]. ML-driven predictive models have shown remarkable success in various applications compared to wet lab methods like directed evolution and traditional computational methods like rational design. However, the incorporation of natural language processing (NLP) techniques and generative models in protein engineering has led to a revolution in the field by improving prediction accuracy, reducing data requirements and enabling the generation of novel and functional proteins. Exploiting NLP techniques have been made feasible by changing the perspective about proteins and finding similarities between human language and protein sequences. For example, both feature an alphabet (20 amino acids in terms of proteins), have hierarchy in the organization, and evolve over time [24]. TAPE [25], UniRep [26], ESM [27] and ProtTrans [28] are among the many successful studies that have applied NLP techniques to learn the dependencies in protein sequences and be able to numerically represent them in fixed vector formats (i.e. embeddings) that are rich in semantics and syntax information. Generative models (based on NLP or pure statistical methods) are also used in different tasks such as improved representation of protein sequences and generation of unforeseen protein sequences in nature. BioSeqVAE [29], ProtGPT2 [30], ProteinGAN [31] and RFdiffusion [32] are examples that generated *de novo* proteins via different model architectures such as variational autoencoder, transformer, adversarial neural networks and diffusion models, respectively.

**Figure 1:**
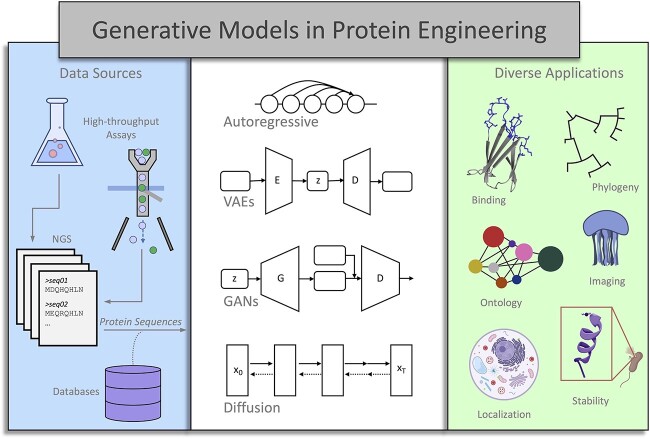
A diverse set of protein engineering applications benefit from the generative and discriminative potential of sequence models. These applications include stability, solubility, bioluminescence, binding capacity, phylogeny, gene ontology and protein localization. The schematic of the sequence models are represented here but their detailed description will be elaborated in their corresponding sections. The autoregressive model forecasts future values based on the previous values in the time series data. VAE is probabilistic modeling architecture that contains an encoder (E) and a decoder (D), compressing high-dimensional input data with the E and reconstructing the data from hidden dimension by D. This architecture along with variational inference techniques will lead to learning given data distribution and generating novel instances. In GANs, G represents a generative model that aims to generate realistic data from noise input, and D is a discriminator that acts as a critic to distinguish the real data from model-generated data. Diffusion models are a relatively new generative model that facilitates the generation of novel samples from a state of maximum randomness (at point X_T_) that is previously generated through the iterative addition of random noise to data distribution. These models have been demonstrated in diverse applications ranging from antibody binding prediction to protein localization prediction tasks in addition to novel protein sequence generation tasks.

Here, we provide a systematic overview of promising neural networks applicable to *protein sequences*. For each architecture, we introduce core mathematical details of the model before describing the implementation of these models towards protein engineering tasks. For each model type, we also provide commentary through selected case studies to describe practical considerations for integrating ML towards protein applications and to illustrate how specific model architectures can be advantageous for a given protein engineering task. The first type to be discussed is language models which have several features that make them strong candidates to be applied for protein sequence data. These techniques can handle variable sequence lengths, and they can track sequence long-term dependencies while maintaining the order of tokens (e.g. amino acid positions). We start with recurrent neural networks (RNNs), then introduce self-attention mechanism (when the model learns to selectively focus on important positions of the input sequence), and finally dive into the transformers and their variants. After discussing transformers, we examine three other generative models in detail: variational autoencoders (VAEs) [33], generative adversarial neural networks (GANs) [34] and diffusion models [35]. While VAE takes a probabilistic approach to learn the training data distribution, GANs use two competing neural networks to generate realistic samples. Diffusion models take a different approach by progressively adding noise to the data until it reaches the prior distribution. Then, new samples can be generated in the reverse diffusion process. Finally, we discuss the current challenges and future perspectives in applying such models to protein engineering. Figure 1 represents the overview of models and applications that are discussed in detail in this study. With this information, we hope researchers are better prepared to integrate these technologies into future investigations.

## SEQUENCE FORECASTING – RNNs & ARs

Given that proteins consist of linear chains of discrete amino acids, the amino acid sequences can be treated as time-series data, with each amino acid acting as a discrete data point for each position (i.e. time point). Because of this attribute, models that forecast future values using past values can be used to sequentially generate amino acids based on previous amino acids in the sequence chain. In this section, we discuss two major models for sequence forecasting: recurrent neural networks (RNNs) and autoregressive models (ARs).

**Figure 2:**
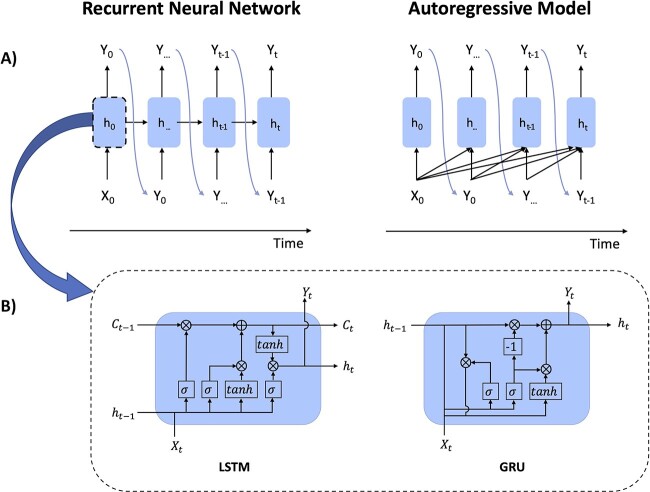
The architecture of generative recurrent neural networks versus autoregressive model. (**A**) An autoregressive model (AR) has a similar structure to a recurrent neural network (RNN). However, while RNN only depends on the current time step, AR utilizes information from the previous time steps as well as the current time step to predict the next token. (**B**) Two important RNN architectures for resolving vanishing gradient problems in training sequence data are LSTM and GRU. These networks contain gates to control the information flow. LSTM contains three gates: input gate, forget gate and output gate and GRU contains two gates: reset gate and update gate. Note that C indicates the cell state, and h is the hidden state in shown architectures.

RNNs are a popular architecture in natural language processing and speech recognition because they hold ‘memory’ by having internal weights that store information from the past that can be updated with every new token processed. There are different types of RNNs including one-to-one, one-to-many, many-to-one, and many-to-many [36]. For protein sequence models, one-to-many RNNs are suitable for sequence forecasting. By giving a starting token to initialize RNNs, the trained model can sequentially produce tokens at the current time step using the output token from the previous time step (Figure 2A). Despite being an architecture well suited for sequential data, some disadvantages of RNNs need to be addressed. During prediction tasks on a given token, RNNs are not able to learn any effects of subsequent parts of the sequence because RNNs process sequences unidirectionally (usually from left to right). This could hurt predictive power because the interactions between amino acids within a protein occur in a three-dimensional space. Bidirectional RNNs (BRNNs) address the issue of unidirectionality and improve prediction performance using two connected layers: one-layer processes sequences in the forward direction and another layer processes sequences in the backward direction [37]. In this way, sequence information is learned from both directions. BRNNs are also successfully applied to unsupervised tasks by enabling their probabilistic interpretation to reconstruct the missing value [38].

Optimizing RNNs while tracking long-term dependencies within sequences can be challenging. Due to the repeated weight matrix multiplication, the weights from early parts of the sequence have a progressively lower influence on the final representation relative to the later parts. The repeated multiplication of small weights results in even smaller weights, causing the gradient vanishing problem in which the gradient eventually reaches a drastically small value. Therefore, RNNs tend to generate biased parameters that capture short-term dependencies, especially when dealing with long sequences. To enable long-term memory, Hochreiter and Schmidhuber introduced Long Short-Term Memory (LSTM) [38], and Chung and colleagues introduced Gated Recurrent Units (GRU) [39] (Figure 2B). Both networks have a gated cell that not only contains multiplication but also addition operations of sigmoid and hyperbolic tangent (*tanh*) functions to regulate the information flow. The sigmoid activation function—which outputs values between 0 and 1—serves as a gate to keep relevant and discard irrelevant information. The information is pertinent for prediction when values are close to 1, and it is kept to future time steps. The tanh activation function regulates values to prevent vanishing and exploding gradients by limiting outputs between −1 and 1.

RNN and its variants have been implemented in protein design tasks for both generative and discriminative applications. For example, a generative LSTM-based model was trained to design *de novo* antimicrobial peptide [40]. The generated sequences elicited higher microbial activity than sampling randomly mutated peptides. In another study, LSTM units were implemented to generate antibody sequences with well-correlated negative log-likelihood and more than 100-fold affinity maturation [41]. Interestingly, an LSTM model trained on only f and y angles of each residue enabled helical protein design [42]. The authors demonstrated that dihedral angles are adequate features to design protein backbones without considering amino acids in a sequence. In another study for predicting protein secondary structures, bidirectional recurrent neural networks with GRU units were used to capture global features within sequence [43].

ARs share a similar structure with RNNs (Figure 2A). Both outputs at time *t* depend on input not only at time *t* but also from earlier time steps. However, RNNs use the hidden state weights from only the most recent time step, whereas ARs use actual inputs from the past to generate future values. AR models, as the name suggests, perform regression tasks over their own lagged variables (i.e. forecasting future values using linear combinations of past values). Protein sequence generation through ARs can be achieved by maximizing sequence likelihood through a tractable probability density function. This objective function is a product of conditional probabilities of tokens at each position that are conditioned on all previous tokens shown as Equation 1, where *X* is the full-length sequence, *x* is each token, *I* is the position number, and *n* is the sequence length. The objective function is decomposed from the joint probability of a full-length sequence using the chain rule of probability and Bayes’ theorem. For each step generation, the features are past tokens, the label is the true token at the current time step, and the loss is the difference between the predicted token and the true token.


(1) \begin{equation*} \boldsymbol{p}\left(\boldsymbol{X}\right)=\prod_{\boldsymbol{i}=\mathbf{1}}^{\boldsymbol{n}}\boldsymbol{p}\left({\boldsymbol{x}}_{\boldsymbol{i}}|{\boldsymbol{x}}_{\mathbf{1}},{\boldsymbol{x}}_{\mathbf{2}},\dots, {\boldsymbol{x}}_{\boldsymbol{i}-\mathbf{1}}\right) \end{equation*}

Theoretically, the space complexity of ARs grows exponentially with forward processing of sequences. This complexity is represented as *O(n^k^)* in big O notation where *k* grows with increasing *n* for a sequence with length of *n*. In practice, ARs use a fixed number of parameters to specify each prior. This reduces the complexity to a polynomial *O(n^c^)* where *c* is a constant. However, this restricts ARs to represent all possible conditional distributions and limits model expressiveness. Lin *et al*.[44] proposed energy-based models and latent-variable autoregressive models as alternatives to alleviate limited distributional modeling of standard ARs.

Recent studies have implemented ARs for protein design. A model with one autoregressive layer paired with generalized logistic regression was used for mutational prediction, contact prediction, and sequence generation of a response-regulator protein family [45]. The negative log-ratio of joint probability of mutant and wildtype were used to indicate single mutational effects and the sum of log probabilities of single mutations were employed as double mutation likelihood for residue-residue contact prediction. The trained model generated sequences that were similar to natural sequences by comparing their principal components. Another study used a dilated convolutional and autoregressive model to model sequential constraints of long nanobodies [46]. They showed that their alignment-free model matches the accuracies of alignment-dependent models in the context of mutation effect prediction, thermostability prediction and fitness predictions for indels. In addition, their model yielded a designed library that contained stable and functional nanobodies with comparable biochemical properties and enhanced diversity to natural nanobody repertoire.

Though RNNs and ARs are powerful, their sequential operation results in linear-time *O(n)* complexity that makes their training time-consuming. Both RNNs and ARs employ supervised learning which helps models optimize with experience and yield high accuracy. However, it increases the chance to overfitting models if the training data is not well-representing the true data distribution. The LSTM and GRU address some limitations of RNNs, but RNNs are still inefficient due to short-term memory and long gradient path. ARs have explicit probability density function to maximize sequence likelihood, but the computation of a series of conditional probabilities requires significant computational resources. The loss of information during training also hinders the overall performance of RNNs and ARs. These issues are significant when considering whether RNNs andARs should be implemented for sequence generation tasks.

### Protein engineering highlights of RNNs and ARs

A comprehensive collection of notable applications of sequence forecasting models in protein engineering is provided in Table 1 below. Following this, a case study is presented to elucidate the details of a selected paper marked in the table.

**Table 1:** Summary of highlighted applications of sequence forecasting models for protein engineering

Protein Engineering Task	Advancements	Model Type	Training Data Source(s)	Year	Ref.
Classification of sequences based on predicted iron sequestration capabilities, protease activity, GPCR activity and p450 activity	Ten diverse, unannotated sequences predicted to exhibit iron sequestration activity were experimentally validated. As measured by accuracy, precision, recall and F1 scores, the RNN model outperforms logistic regression and random forest models.	RNN	UniProt	Jan. 2017	[48]
Predict solvent accessibility.	8.8% mean absolute error and 74.8% Pearson’s correlation coefficient value for predicting solvent accessibility were observed.	Stacked Bidirectional LSTMs	PISCES Database	May 2018	[43]
Structural class prediction.	Using a low-dimension feature space (18-D), classification accuracies ranging from 84.2% to 95.9% were observed.	RNN	PDB25, FC699, 640, 498, 277 and 204	June 2021	[49]
Novel artificial protein sequence generation.	Libraries of artificially generated monobodies mutases were experimentally validated	DCA	1259 natural monobodies mutase sequences	July 2020	[50]
Antibody binding pocket prediction.	Outperformed CNN/RNN models and random-forest models	Bidirectional LSTMs	Structural Antibody Database (SabDab)	Dec. 2021	[47]^a^

^a^See discussion below for a more detailed case study.

#### Prediction of antibody paratope with bidirectional LSTMs

A deep learning model for predicting the antigen binding sites of antibodies was developed through the implementation of bidirectional LSTMs in a transformer neural network (discussed in Section Sequence Design with Attention Mechanism: Transformer-Based Language Models) [47]. The DeepANIS (Antibody Interacting Site) model was able to elucidate the relationships among residues of the loop sequences of complementarity determining regions (CDRs) of a given antibody (https://github.com/HideInDust/DeepANIS). Trained on only 277 antibody–antigen complexes from the Protein Sequence Culling Server (PISCES) database, the authors demonstrated the ability of a transformer neural network using bidirectional LSTMs to outperform alternative CNN-based and random-forest-based models for paratope prediction. This architecture also enabled the developers to perform these predictions using the concatenated CDR loop sequences of a given antibody as the only input. Alternative models require either the CDRs to be provided as separate sequences for each CDR loop or additional information about the antigen or antibody.

## SEQUENCE DESIGN WITH ATTENTION MECHANISM: TRANSFORMER-BASED LANGUAGE MODELS

Transformer models consist of a specific neural network architecture that transforms the input sequences to output sequences using a series of operations (e.g. matrix multiplications, scaled dot product attention and feed forward neural network). Transformers have given rise to various sequence-to-sequence models such as machine translation, question answering (chat bot) and text summarization. Their specific design enables parallel operation (constant-time *O(1)* complexity), resulting in faster and more efficient performance than ARs and RNNs (linear-time *O(n)* complexity). This parallelization improves uniform learning across each position of a sequence by eliminating short-term dependencies that disproportionately weigh later parts of the sequence compared to earlier parts.

The original transformer introduced by Vaswani *et al.*[15] consists of an ‘encoder’ that encodes a complete sentence to a representation and a ‘decoder’ that decodes a target sentence with the contextual representation (Figure 2). Both the encoder and decoder contain multiple self-attention and feed forward neural network units. Self-attention is a key component in transformers that enables the model to know which tokens are important in processing the given token (e.g. epistatic interaction in protein sequences). The feed forward networks are then used for adding non-linear operations to the network in training. Note that the order information of tokens gets lost due to parallel computing. Thus, transformers have an additional embedding called positional encoding, which encodes the exact position of tokens using as many sinusoidal functions as embedding dimensions.

To train a transformer to translate text from language A to language B, the encoder uses language A as an input to generate a representation. The decoder uses language B as an input and combines this with encoder-generated representation to learn the correct mapping of words in two distinct languages. Similarly, protein-specific *de novo* drug design can be treated as a translational problem [51]. The authors used a transformer as a biological translator to generate novel molecule binders given amino acid sequences only. For a question-answer task, the encoder input is the question, and the decoder input is the answer. Through a connected encoder and decoder, the transformer learns to give an answer based on a specified question. Protein–protein interactions are analogous to question-answer pairs in syntax. This strategy was utilized to generate signal peptides via available organism data in Swiss-Prot [52]. Their experimental results showed that the generated peptides are functional and diverse.

**Figure 3:**
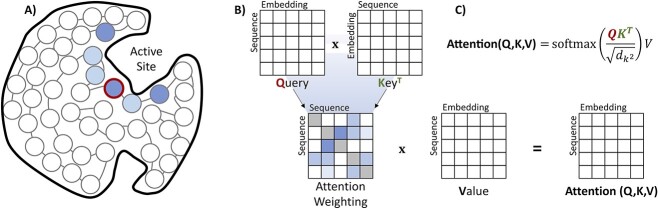
Visualization of attention mapping and attention computation. (**A**) Based on the protein fold, amino acids in different positions have varied epistitatic effects on each other. The highlighted circle refers to a query amino acid in a protein active site. The color gradient shows how attention can capture the influence of other amino acids (tokens) on the queried token. (**B**) Attention computation requires three components: key, query and value. By calculating scaled dot-product attention scores, the model chooses which areas of the sequence it needs to prioritize for the prediction task.

As noted in Figure 3, transformers capture the influence of other tokens (e.g. through epistasis) on the query token with a self-attention mechanism. The self-attention computation starts with three inputs: query, key, and value; analogous to those in retrieval systems. When retrieving an item, the machine takes a request (query) against a list of descriptions of items (keys) and returns top matches (values). In protein chains, we retrieve attention from a sequence first by having queries (amino acid requests) multiplied with transposed keys (amino acid identities) to obtain attention weighting. Then, the scaled and normalized attention weighting is multiplied with values (amino acid representations) to obtain attention (Figure 3). Often, the attention layer is split into several heads in parallel to capture attention from different subspaces. The multi-head attention layer combined with a fully connected feed-forward network with layer normalization in between builds an attention block; these blocks are then combined to form the encoder. Since the inputs (query, key and value) of the encoder are from the same sequence, it generates a self-attentive representation of that sequence. The attention block of the decoder has an additional layer: masked multi-head attention layer, which is placed before the multi-head attention layer. The inputs of the masked attention layer are from the decoder, meaning that it is self-attentive. The inputs of the following attention layer are from both the encoder and decoder (query from decoder; key and value from encoder), meaning that it is cross-attentive. Due to this self- and cross-attention mechanism, the decoder generates a target sequence considering both the encoder and the decoder.

Attention mechanism has been applied to understand the semantics and syntax of protein language. It has been implemented between sets of gene ontology terms to predict protein–protein interactions [53]. This mechanism has also been employed with a convolutional neural network (CNN) to predict protein contact [54, 55]. Similarly, in another report, CNN with attention mechanism improved protein-drug interaction prediction [56]. CNN was also used to obtain feature metrics of the proteins and ligands. Attention mechanism was then implemented to assign weights to each atom or amino acid. Their model evaluation of benchmark datasets showed improvements compared to previous baselines.

**Figure 4:**
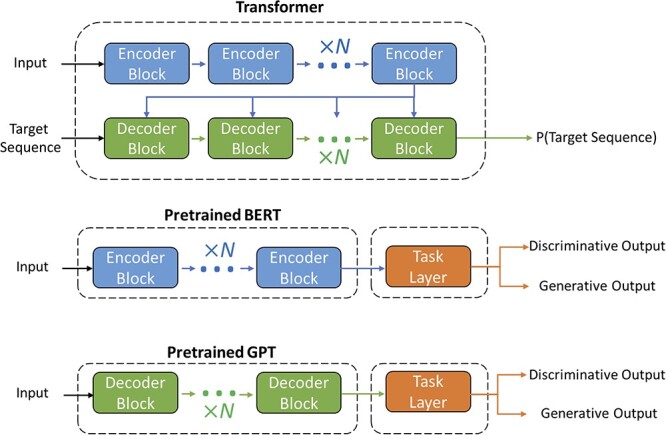
Architecture overview for the transformer and two important transformer-based language models: Bidirectional Encoder Representation from Transformers (BERT) and Generative Pre-Training (GPT). The transformer utilizes an encoder-decoder method for handling language tasks. However, BERT uses encoder blocks only and GPT only includes decoder blocks. The difference in their architecture is mainly due to their training objective. In pretraining, BERT takes a bidirectional approach while GPT is based on an autoregressive method.

The transformer decoder is autoregressive by nature, owing to its masked self-attention layer. By masking out the attention of future tokens, the decoder decodes target sequences to infer the attention of past tokens. This is achieved through the addition of attention weighting and a mask matrix, whose upper triangular is filled with negative infinity and lower triangular is filled with zeros. While the decoder is autoregressive during testing, it is non-autoregressive at training time. During training, the decoder generates tokens at all time steps simultaneously, not relying on tokens at previous time steps. The autoregressive attribute of the decoder allows transformers to be used in generative applications. Figure 4 represents the overall schematic of transformer architecture and its variants.

The ability of transformers to generate text representation prompted the invention of transformer-variant models: Bidirectional Encoder Representation from Transformers (BERT) and Generative Pre-Training (GPT) [57, 58]. They can generate meaningful representations which can be used for downstream, task-specific modeling (e.g. named entity recognition, question-answering and text generation). Pre-training models with a large corpus (unsupervised training) followed by fine-tuning with task-specific objectives (supervised training) result in improved performance in different language modeling tasks. Note that BERT and GPT also have shown good performance in few-shot (i.e. when there are only few labeled data are available) and zero-shot (i.e. when the model generalizes to the new task with no further data for training) settings [58–60]. The architecture of BERT is composed of layers of original transformer encoders. The pre-training of BERT uses an approach prevalent in masked language modeling (MLM). By masking out tokens in an input sentence, the models are trained to predict masked tokens using their context. This is achieved by minimizing the cross-entropy loss between **masked** and actual tokens. This context-dependent training makes representation bidirectional, which is why BERT is a popular architecture for representation learning. In contrast, GPT comprises a series of transformer decoder architecture without the multi-head cross-attention layer due to the absence of encoders. In contrast to BERT’s MLM, GPT uses a casual language modeling (CLM) approach that predicts masked tokens, only considering tokens on the left side. By left-shifting each token in input sequences, GPT does not have access to the actual token that is going to predict. Therefore, the representation generated from GPT pre-training is unidirectional and self-attentive, making it a popular model for text generation. By having the task layer directly working on pre-trained representation, the number of layers, learnable parameters and training time are reduced. The training of task layer occurs simultaneously with the fine-tuning of pre-trained models to improve the compatibility between a representation and a given task. Based on the architecture of task layers, they handle either sequence-to-sequence (sequence generation) or sequence-to-scalar (sequence classification) tasks.

In general, BERT is not optimal for text generation, and GPT is limited to only unidirectional interactions. Lewis *et al.*[61] proposed bidirectional autoregressive representation from transformers (BART) that combines characteristics of BERT and GPT to execute sequence-to-sequence denoizing. The BART encoder learns from corrupted sequences that introduce noises to the model through masking, insertion, deletion, infilling, permutation and rotation. The BART decoder learns to reconstruct original sequences autoregressively. The encoder and decoder work together to recognize and remove intentionally added noise. Hence, BART is a useful architecture for sequence noise reduction and feature extraction. Another challenge in training is capturing long-term dependencies for sequence data whose length is much greater than its embedding dimension. The BART-derived Performer model was proposed to reduce the cost of training the attention mechanism which scales linearly instead of quadratically with sequence length [62]. This model presents an unbiased estimation of a regular attention matrix with which the estimation is uniformly convergent.

### Protein engineering highlights of transformer-based language models

Table 2 below presents a wide range of impactful applications of transformer-based language models in protein engineering. Following this table, two case studies selected from the table are discussed.

**Table 2:** Summary of highlighted applications of transformer-based models for protein engineering

Protein Engineering Task	Advancements	Model Type	Training Data Source(s)	Year	Ref.
Protein sequence language modeling	Approached protein sequence modeling as a language translation task using one RNN to encode the protein sequence and another RNN to decode the previously encoded sequence.	Transformer	CAFA3	Oct. 2017	[64]
DNA-binding protein prediction	Incorporated sequence context with attention mechanism to improve prediction performance over random-forest, RNN, and support vector machine models.	CNN-Bidirectional RNN	UniProt	Nov. 2019	[65]
Signal peptide sequence generation	A diverse library of 53 artificially generated signal peptides were generated and validated in *Bacillus subtilis*.	Transformer	Swiss-Prot	Aug. 2020	[52]
Novel sequence generation for enhanced GB-1 variants.	Used Gibbs Sampling to generate new sequences from BERT language model.	BERT	GB1	Jan. 2021	[66]
Homology, solubility, subcellular localization, stability, fluorescence, secondary structure, and topology prediction	Demonstrated a pre-training strategy that incorporates sequence data with structural information which improved performance on protein fitness prediction tasks compared to similar-size language models.	Bidirectional RNN	Pfam	Sept. 2021	[63]^a^
Variant Effect Prediction	Introduced a new approach to incorporating specialized attention heads and sequence context information	Transformer	UniRef100	June 2022	[67]
Novel sequence generation	Generated novel protein sequences that mimic properties of natural proteins (e.g. stability, dynamics). Pre-trained model enables rapid, accessible sequence generation on desktop machines.	Transformer	UniRef50, Swiss-Prot	July 2022	[30]^a^
GFP fluorescence intensity, stability	Introduced key design constraints to Transformer model architecture in their regularized latent space optimization (ReLSO) approach to protein sequence modeling.	Transformer	GB1, Gifford, GFP, TAPE	Oct. 2022	[68]

^a^See discussion below for a more detailed case study.

#### Pre-training of deep bidirectional protein sequence representations with structural information

The pre-training scheme PLUS was able to outperform leading pre-training models that are based solely on language models (e.g. UniRep, P-ELMo) by integrating protein-specific structural information with amino acid sequence data (https://github.com/mswzeus/PLUS) [63]. Structural information was obtained from protein family labels among the Pfam dataset. This provided a more accurate and less computationally intensive route compared with using sequence similarity to predict protein function. Additionally, masked language modeling is performed in a similar manner used in BERT to extract syntactic and semantic information. In this study, an informative comparison was made wherein PLUS was used to pre-train bidirectional RNN (PLUS-RNN) and Transformer (PLUS-TFM) architectures. Despite much of the literature indicating that attention-based models are superior, the PLUS-RNN architecture was found to be advantageous over the PLUS-TFM in this study due to the RNN-based implementation more accurately capturing local amino acid sequence motifs. For PLUS-RNN, bidirectional representations of amino acid sequences were used to capture context in both the right-to-left and left-to-right directions. In doing so, the PLUS-RNN model achieved higher performance than similarly sized transformer in protein-level classification and regression tasks and amino acid-level classification tasks. Higher performance was observed even against the leading task-specific models in predicting homology, stability, fluorescence and transmembrane residues.

#### ProtGPT2 is a deep unsupervised language model for protein design

ProtGPT2 is an autoregressive Transformer model capable of generating highly diverse protein sequences [30]. Among the generated sequences, amino acid propensities and fraction of disordered regions are consistent with proteins found in nature, yet the generated sequences are highly distinct from natural proteins. ProtGPT2 provides a useful platform for finetuning based on a particular protein family, function, or fold of interest. Using a Transformer decoder model with byte-pair encoded input sequences enabled self-supervised training on nearly 50 million unlabeled protein sequences from UniRef and Swiss-Prot. With 738 million parameters, ProtGPT2 allows users to generate novel sequences in mere seconds on a desktop computer (https://huggingface.co/nferruz/ProtGPT2).

## PRE-TRAINED LANGUAGE MODELS & EMBEDDINGS

Transfer Learning (TL) is a ML technique to transfer useful knowledge learned from a source domain to another related domain (i.e. target domain). This is particularly useful when there is a lack of labeled data in the target domain and obtaining labeled data is time-consuming and costly. Inductive learning, transudative learning and unsupervised learning are specific transfer learning approaches for different applications. Inductive TL improves the target predictive function via the information learned from the source domain prediction task. Note that both the domain and tasks are different but related. Transudative TL aims to improve prediction in target tasks using the learned knowledge from the source domain. However, the learning tasks need to be the same while domains are different. For unsupervised TL, the target domain prediction function still benefits from the source domain when the learning tasks are not the same and there is no labeled data in both the source and the target domain[69].

The use of TL in protein engineering applications can increase the efficiency and generalizability of the downstream tasks via transferring the domain knowledge learned in pretraining to the prediction task. One highly explored and successful application of TL in protein engineering is the use of pretrained language models for predicting protein properties (e.g. thermostability, kinetic activity, binding affinity and disordered regions) from its sequence. These models are trained over a large number of unlabeled sequences in protein databases such as UniProt [70], UniRef [71] and SRA [72]. With NLP techniques such as masked-token prediction and next-token prediction, these models extract useful information from their training data to be used in downstream tasks. Note that the trained model can either be directly used or its information can be extracted to a fixed-size continuous vector (i.e. an embedding). These embeddings are unique for each input sequence, and they contain structural, evolutionary, statistical and biophysical information about the proteins. This is considered a breakthrough in ML-guided protein engineering tasks where pretrained models alleviate the lack of data and improve performance of ML models. Unified representation (UniRep) is among the early pretrained models which was trained via an mLSTM model over 25 million sequences to distill biophysical and evolutionary information of proteins and represent it in a fixed size representation. The UniRep model has shown generalizations to distant regions of fitness landscape in addition to low number data requirements for viable predictions [73].

### Protein engineering highlights of pre-trained Language Models & Embeddings

Table 3 contains a collection of highlighted applications of pre-trained language models and embeddings in protein engineering, along with an added case study for further understanding.

**Table 3:** Summary of highlighted applications of transfer learning & embeddings for protein engineering

Protein Engineering Task	Advancements	Model Type	Training Data Source(s)	Year	Ref.
Stability Prediction, fitness (GFP brightness) prediction	Introduced RNN-based unified representation (UniRep) to improve efficiency in protein engineering tasks.	Transformer	UniRef50	Dec. 2019	[26]
Secondary Structure, Subcellular localization, and solubility prediction	Developed the SeqVec model of protein sequences that represents sequences as continuous vectors to predict biophysical and biochemical properties.	ELMo	UniRef50	Dec. 2019	[75]
Secondary Structure, Contact, Homology, Fluorescence, and Stability Prediction	Demonstrated the usefulness of multi-task benchmarks like TAPE to evaluate protein transfer learning models.	Transformer	CB513, CASP12, TS115, avGFP, ProteinNet, SCOP 1.75	Dec. 2019	[25]
Protein Family Classification, Protein Interaction Prediction	Using the training procedure developed by RoBERTa, PRoBERTa offers a more generalized pre-training framework that outperformed in protein interaction prediction tasks.	Transformer	UniProtKB/Swiss-Prot	Jun 2020	[76]
Protein folding, binding site, and substitution matrix prediction	Demonstrated how attention can capture protein features from BERT-based models for protein engineering tasks.	Transformer	Pfam, BFD, UniRef100	March 2021	[77]
Secondary Structure, Subcellular Localization, and Solubility Prediction	Demonstrated the usefulness of pre-trained embeddings for various prediction tasks without requiring the use of MSAs.	ARs	UniRef, BDF	July 2021	[28]
Secondary Structure, Contact, Homology, Fluorescence, and Stability Prediction	Improved protein representations by incorporating pairwise masked language model to encode co-evolutionary information.	Transformer	Pfam, UniRef50	Oct. 202 1	[78]
Sequence Profile Construction	Performed sequence profile reconstruction with the pre-trained ProtAlbert for sequences with limited homology to database sequences.	Transformer	UniRef, BDF	Aug. 2022	[79]
Structure Prediction	Despite not requiring the use of MSAs, ESM Fold—trained on UniRef sequences—offers rapid structure generation from a single input protein sequence.	Transformer	UniRef50 and UniRef90	March 2023	[26, 74]^a^

^a^See discussion below for a more detailed case study.

#### Unsupervised learning on 250M protein sequences results in deriving biological insights

BERT and GPT are versatile if trained appropriately and have been successfully implemented in the protein sequence domain. Rives *et al.*[27] trained BERT with 250 million protein sequences to generate representation that contains biological properties. They employed downstream tasks including remote homology, linear projection, secondary structure prediction and contact prediction to showcase the information-rich representation from their deep contextual language model, evolutionary scaling modeling (ESM) (https://github.com/facebookresearch/esm). They also showed how sequence diversity and model size influence the model performance. To enable transfer learning of a model to a new task with no additional supervision, extended ESM architectures such as ESM-1v and ESM2 were proposed for variant effect prediction (i.e. mapping sequence changes to functional changes) and capturing high-resolution structural features, respectively[74].

There are several attempts to model protein sequences via language model techniques to apply the learned information about protein sequences to protein engineering tasks; some of the most successful ones are listed above. Embedding methods can alleviate the lack of labeled data and improve generalization. In addition, training over self-supervised methods with more parameters leads to capturing more nuanced information about the language of proteins [74]. While transfer learning has shown great promise in protein engineering applications, there is a need for a deeper understanding of what information is learned in pretraining and transferred to the downstream prediction tasks [80]. For example, some studies have observed similar or superior performance for protein fitness prediction without the use of embedding methods [81, 82].

## PROBABILISTIC MODELING OF SEQUENCE–VAEs

**Figure 5:**
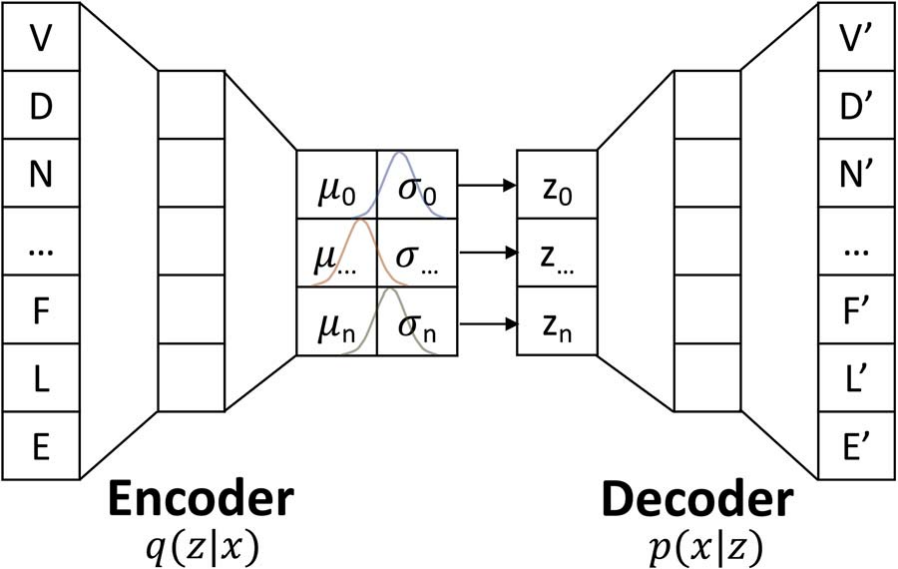
Sequence probabilistic modeling is feasible via encoder-decoder architecture and variational inference. A parameterized distribution function is determined for the given sequence data in which new sequences get generated by sampling from the learned distribution. VAE architecture consists of an encoder q(z|x) to map the input from x to z and a decoder p(x|z) to map the data from z back to x.

Unlike transformers that treat protein sequences as a language, variational autoencoders (VAEs) treat sequences as a parameterized multivariate distribution [83]. VAE architecture consists of taking high-dimensional data, reducing it to a low-dimensional representation (encoder) and then reconstructing the representation into the original dimensionality as the input data (decoder) (Figure 5). This encoder-decoder bottleneck structure is also a hallmark of standard autoencoders (AEs). The latent space representation of AEs is a fixed length vector where each value (dimension) is associated with a single learned feature from data. However, the latent representation of VAEs are probability distributions (which are continuous and smooth) for each data attribute. By randomly sampling a vector from latent state distributions, the VAE decoder acts as a generative model that can generate new data instances (e.g. novel protein sequences). The VAE encoder is a recognition model with the ability to recognize statistical distributions that describe variations in data.

**Figure 6:**
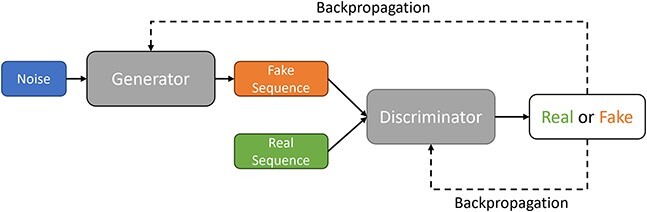
GANS architecture for generating sequence data; a model that learns to sample from the given data distribution which contains two separate and opposed networks: generator and discriminator. The generator aims to generate synthetic data from noise which can’t be distinguished from the real data by the discriminator. The discriminator on the other hand gets optimized to identify synthetic data from the real data. Evolving together, the model finally will be able to generate samples very similar to the real training data.

The latent representation of VAEs is forced to be continuous and smooth by training the encoder to output pairs of mean and standard deviation (probability distributions) which are subsequently sampled by the decoder. Compared with discrete variable representation of AEs, the continuous distribution representation of VAEs allows the decoder to learn that both a single value and its nearby values refer to the same class. Accordingly, representations of the same class are clustered together as a distribution in latent space, and nearby representations have similar reconstructions. The gap between classes in latent space is troublesome, as the decoder has no training data to learn features of those space. Therefore, VAEs incorporate a term in the loss function, the Kullback–Leibler (KL) divergence, which measures how one distribution is different from another. By minimizing the KL-divergence between the learned latent distribution and a prior distribution (e.g. Gaussian), VAEs regularize the latent space. This regularization promotes a continuous latent space which allows VAEs to interpolate values smoothly from one class to another. The reconstruction loss encourages the formation of data points similar to the original input and KL-divergence regularizes the latent space. This incorporation results in a well-structured and information-rich latent space where VAEs can sample from.

Instinctively, for latent variables *z* that generates observation *x*, we maximize data likelihood $p(x)$ by maximizing $\int p\left(x|z\right)p(z)\ dz$. However, this integral is intractable and cannot be directly optimized. Instead, VAEs use a derivative data likelihood to model $p(x)$ with encoder distribution $p\left(z|x\right)$, decoder distribution $p\left(x|z\right)$, and latent variables $p(z)$. The posterior distribution, $p\left(z|x\right)$, which refers to attributes of latent variables from observation is also intractable, but we can apply variational inference to approximate this density function [84]. By defining a tractable encoder distribution $q\left(z|x\right)$ and minimizing KL-divergence between $p\left(z|x\right)$ and $q\left(z|x\right)$, we obtain the objective function of VAEs with derivation shown below:


(2) \begin{equation*} {D}_{KL}\left[q\left(z|x\right)\mid \left|p\left(z|x\right)\right]={D}_{KL}\right[q\left(z|x\right)\mid \left|p(z)\right]-{E}_q\left[\log p\left(x|z\right)\right]+\log p(x) \end{equation*}

(3) \begin{align*} & \log p(x)-{D}_{KL}\left[q\left(z|x\right)\mid \big|p\left(z|x\right)\right]\nonumber\\&\qquad ={E}_q\left[\log p\left(x|z\right)\right]-{D}_{KL}\left[q\left(z|x\right)\mid \big|p(z)\right]= ELBO \end{align*}

(4) \begin{equation*} \log p(x)\ge ELBO \end{equation*}

Since D_KL_[*p*(*z*|*x*)||*q*(*z*|*x*)] is intractable and KL divergence is always positive, we can maximize tractable Evidence Lower-Bound (ELBO) in order to maximize log data likelihood. Within this objective function, ${E}_q\left[\log p\left(x|z\right)\right]$ corresponds with the reconstruction loss, and ${D}_{KL}\Big[q\left(z|x\right)\mid \left|p(z)\right]$ corresponds KL-divergence loss mentioned in previous paragraph.

Several VAE-derived models have been developed to address common issues like attribute entanglement and posterior collapse. Higgins *et al.*[85] proposed Beta-VAEs to facilitate learning of the disentanglement of data attributes. By introducing a hyperparameter $\mathrm{\beta}$ that penalizes KL divergence loss, the latent representation is forced to adjust the trade-off between reconstruction and regularization. Razavi *et al.*[86] proposed a method for preventing a common issue in training VAEs, posterior collapse. Posterior collapse happens when the posterior fails to capture the true posterior of the latent variables, and the model gets ineffective in generating diverse and high-quality samples. Their proposed method, delta-VAE, restricts parameters of the posterior to establish minimum KL divergence between prior and posterior.

### Protein engineering highlights of VAEs

Explore Table 4 for an overview of how VAE models have been implemented for protein engineering applications.

**Table 4:** Summary of highlighted applications of VAE models for protein engineering

Protein Engineering Task	Advancements	Model Type	Training Data Source(s)	Year	Ref.
Metallo protein sequence generation and metal binding site prediction	Generated novel metallo proteins that feature copper and calcium binding sites	VAE	UniRef90	Nov. 2018	[88]
Novel T cell receptor sequence generation	VAE model was able to estimate cohort frequency, learn VDJ recombination regulation, generalize unexplored sequence space, and generate novel T cell receptor sequences	VAE	Adaptive Biotech immuneAccess	Sept. 2019	[87]*
Protein dynamics prediction	Introduced a novel approach to unsupervised learning to train VAEs	VAE	MoDEL	May 2020	[89]
Novel protein sequence generation	Implemented “Deep Exploration Nertwork *“*architecture to generate novel sequences with polyadenylation sites, splicing sites, transcription enhancer binding sites, and enhances GFP fluorescence	VAE	MPRA, APA, avGFP	July 2020	[90]
Novel luciferase sequence generation	Novel luciferase sequences generated from VAE model resulted in increased solubility while maintaining bioluminescence.	VAE	InterPro	Feb. 2021	[91]
Novel protein sequence generation	Proposed a new evaluation metric in their comparison of Potts, VAE and site-independent generative models.	VAE	UniProt/TREMBL, Pfam	Nov. 2021	[92]
Ancestral sequence reconstruction	Demonstrated the ability to capture higher-order epistatic effects through VAE-generated phylogenetic trees	VAE	CFP, EvolveAGene4 Simulations, PFAM	Feb. 2023	[93]

^a^See discussion below for a more detailed case study.

#### Deep generative models for T cell receptor protein sequences

Davidsen *et al.*[87] demonstrated the capability of simple VAE models to generate T-cell receptor (TCR) sequences with similar characteristics to real sequences (https://github.com/matsengrp/vampire/). Rather than modeling the probability of a given sequence to undergo V(D)J recombination that approaches the properties of the mature TCR repertoire, the architectures of VAEs enables the direct modeling of the distribution of the mature TCR repertoire. In addition to generating novel TCR sequences, the VAE-based models were able to predict the frequency of a TCR in a given cohort and learn the rules of V(D)J recombination. The training data of TCR sequence repertoires were sourced from Adaptive Biotechnologies’ ImmunoSEQ assay. Despite some requiring <100 lines of Python code, these simple VAE models were found to outperform previous models that implemented more complicated graphical models that mimic the biological process of V(D)J recombination.

## SEQUENCE GENERATION THROUGH MinMax GAMING–GANs

Up to this point, we discussed generative models that use explicit probability density functions. RNNs and Ars have a tractable function, and VAEs have an approximate function to maximize likelihood. Here, we turn to generative adversarial networks (GANs), an implicit probabilistic model that directly generates new data instances by defining a stochastic procedure [34].

GANs employ a two-player game approach to replicate training data distributions without assumptions about their priors. One player is the generator network, and the discriminator network is the other player. The objective of the generator is to generate realistic data instances from random noise to fool the discriminator. On the other hand, the objective of the discriminator is to distinguish real and fake data from the training set and the generator, respectively (Figure 6). By having these two networks competing, the generator learns to generate (fake) data that is close to the (real) training samples, and the discriminator provides feedback to the generator for improvement. This approach allows two networks to evolve with each other so that, ideally, the generator can generate synthetic samples that are indistinguishable from real samples. To train two networks jointly, GANs have a minmax objective function shown below:


(5) \begin{align*} \underset{\theta_G}{\min}\underset{\theta_D}{\max }V\left(D,G\right)=&{E}_{x\sim{p}_{data}}\left[\log{D}_{\theta_D}(x)\right] \nonumber \\&+{E}_{z\sim p(noise)}\left[\log \left(1-{D}_{\theta_D}\left({G}_{\theta_G}(noise)\right)\right)\right] \end{align*}

Minmax function can be interpreted as a function that minimizes the loss that the opponent maximally gives. In GANs, the generator with parameters ${\theta}_G$ wants to minimize the objective value $V\left(D,G\right)$ such that the probability of the discriminator output fake data ${D}_{\theta_D}\left({G}_{\theta_G}(noise)\right)$ is close to 1. This indicates that the generator successfully fooled the discriminator by classifying fake data to real. Conversely, the discriminator with parameters ${\theta}_D$ aims to maximize the objective value such that the probability of the discriminator output real data ${D}_{\theta_D}(x)$ is close to 1, and the probability of the discriminator output fake data ${D}_{\theta_D}\left({G}_{\theta_G}(noise)\right)$ is close to 0. The training with this minmax function is equivalent to have the generator performing gradient descent on term $\log \left(1-{D}_{\theta_D}\left({G}_{\theta_G}(noise)\right)\right)$ and the discriminator performing gradient ascent on $V\left(D,G\right)$. However, the generator of GANs is likely to get stuck in the early stage of training (caused by small gradients) when generated samples are easy to be classified as fake. In practice, the generator performs gradient ascent on term $\log \left({D}_{\theta_D}\left({G}_{\theta_G}(noise)\right)\right)$ instead of gradient descent on term $\log \left(1-{D}_{\theta_D}\left({G}_{\theta_G}(noise)\right)\right)$. In this manner, GANs have steep gradient to drive learning by maximizing the likelihood of the discriminator being wrong instead of minimizing the likelihood of the discriminator being correct.

Developing a loss function for GANs that leads to more stable and better learning is still an active research area. Arjovsky *et al*.[94] proposed Wasserstein Loss in which the discriminator maximizes ${D}_{\theta_D}(x)-{D}_{\theta_D}\left({G}_{\theta_G}(noise)\right)$, and the generator maximizes ${D}_{\theta_D}\left({G}_{\theta_G}(noise)\right)$. This means that the discriminator is not a classifier but a ‘critic’ that maximizes the difference between proxy number of fake and real data, while the generator maximizes the output of discriminator given generated (fake) data. Usually, the trained discriminator is discarded, and the trained generator is kept for new data generation.

### Protein engineering highlights of GAN models

Table 5 illustrates a selection of key GANs models applications in protein engineering, with an added case study for deeper analysis.

**Table 5:** Summary of highlighted applications of GAN models for protein engineering

Protein Engineering Task	Advancements	Model Type	Training Data Source(s)	Year	Ref.
Antimicrobial peptide sequence generation	Implemented a GAN model to generate novel antimicrobial peptides	GAN	UniProt	Feb. 2019	[96]
Target-drug binding affinity prediction	Used unlabeled data to train GAN model in a semi-supervised manner	GAN	[97], KIBA	Jan. 2020	[98]
Antibody design	Experimentally validated GAN-generated antibody sequences after initial phage display library screening	GAN	Observed Antibody Space Repository	April 2020	[99]
Ancestral sequence reconstruction	Performed ancestral sequence reconstruction on H3N2 influenza proteins and predicted the evolution of these proteins to improve pathogen forecasting	GAN	NCBI Influenza Virus Resource	Aug. 2020	[100]
Synthetic data generation, gene ontology prediction tasks	Improved gene ontology prediction with GAN model by creating synthetic feature samples	FFPred-GAN	modENCODE	Sept. 2020	[95]*
Gene ontology classification	Used WGAN to improve gene ontology term correlation performance	WGAN	SwissProt	Feb. 2021	[101]
Novel malate dehydrogenase sequence generation	Experimentally validated novel malate dehydrogenase sequences with up to 106 mutations	GAN	UniProt	April 2021	[31]

^a^See discussion below for a more detailed case study.

#### GAN architecture enables the generation of synthetic samples to improve training

Data augmentation with high-quality synthetic sample data points can help overcome the challenges of developing models that predict protein function. Wan and Jones[95] demonstrate the ability to generate these high-quality synthetic protein feature samples using their GAN-based FFPred-GAN. In addition to using the FFPred model to determine protein biophysical information from protein sequences, FFPred-GAN implemented a WGAN with gradient penalty to learn the distribution of the training protein data set distribution. FFPred-GAN enabled significantly higher accuracy in all the three domains of the gene ontologies domains (i.e. cellular component, molecular function and biological process) without demanding significant computational resources to generate both negative and positive synthetic samples (https://github.com/psipred/FFPredGAN).

## DIFFUSION MODELS

Diffusion is a recently developed and rapidly ascending model in the generative AI domain and has shown competitive performance with established benchmarks. This novel method offers better distribution convergence and more diversity in the generated samples. The diffusion model’s underlying principle is adopted from non-equilibrium thermodynamics in which the diffusion process increases the system’s entropy, driving it towards a state of maximum randomness [35]. Therefore, in the context of generative modeling, diffusion models can gradually transform noisy signals into coherent data structures (i.e. reversing the noise). These models have shown promising results in image synthesis, image inpainting (i.e. filling missing regions in images) and text generation. For example, Dall-E2 [102], a text-to-image framework generated by OpenAI, incorporates a diffusion model during training to generate realistic and high-quality images. Their model resulted in up to four times improvement in resolution compared to the original Dall-E trained with GPT3 architecture [103].

Effective training in generative diffusion models requires a detailed understanding of its main components and foundational concepts. In this section, we describe the core concepts, models, and the main mathematical formulations that have been used for training the diffusion models. Finally, we examine the evolutionary improvements of these models since their introduction in 2015. The forward diffusion process is the transition from data distribution to a prior distribution (e.g. isotropic Gaussian). This is a Markov chain process, and each step only depends on its previous step (i.e. progressively adding noise). For example, we can generate a noisy image at $t=1$ by adding a small amount of Gaussian noise to the pixel values for the image at $t=0$, repeating this process for subsequent time steps until the data distribution transforms into a prior distribution. The forward diffusion step parametrization can be shown below:


(6) \begin{equation*} \mathrm{q}\left({\mathrm{x}}_{\mathrm{t}}|{\mathrm{x}}_{\mathrm{t}-1}\right)=\mathcal{N}\left({\mathrm{x}}_{\mathrm{t}};\sqrt{1-{\mathrm{\beta}}_{\mathrm{t}}}\left({\mathrm{x}}_{\mathrm{t}-1}\right),{\mathrm{\beta}}_{\mathrm{t}}\mathrm{I}\right) \end{equation*}

(7) \begin{equation*} \mathrm{q}\left({\mathrm{x}}_{1:\mathrm{T}}|{\mathrm{x}}_0\right):= {\prod}_{t=1}^Tq\left({x}_t|{x}_{t-1}\right):= {\prod}_{t=1}^T\mathcal{N}\left({x}_t;\sqrt{1-{\beta}_t}\left({x}_{t-1}\right),{\beta}_tI\right) \end{equation*}

where *t* is the time step and it ranges from 1 to *T*, ${\mathrm{x}}_0$ is the instance sampled from the true data distribution, ${\mathrm{\beta}}_t$ is the variance scheduler, and *I* is the identity matrix. Given the equations above, the conditional probability distribution of each step given the previous step is assumed to be a conditional Gaussian distribution with mean $\sqrt{1-{\mathrm{\beta}}_t}\left({x}_{t-1}\right)$ and variance ${\mathrm{\beta}}_tI$. Also, the noised image distribution can be directly obtained at each timestep using a reparameterization trick in a closed form [104]. The Backward Diffusion Process represents the challenging task of transforming the noised distribution to the data distribution. Once accomplished, new data instances can be generated by sampling from the noise distribution. In the backward diffusion process, the model starts with pure Gaussian noise and in each step learns the Gaussian transition parameters with the aid of a parametrized model (e.g. neural networks). Note that this network should have a similar input and output dimension (e.g. UNET [105] architecture). The backward step parametrization is represented in Equation 8 and Equation 9.


(8) \begin{equation*} {p}_{\mathrm{\theta}}\left({x}_{t-1}|{x}_t\right)=\mathcal{N}\left({x}_{t-1};{\mu}_{\mathrm{\theta}}\left({x}_t,t\right),\sum_{\theta}({x}_t,\mathrm{t})\right)\end{equation*}

(9) \begin{equation*} {p}_{\mathrm{\theta}}\left({x}_{0:T}\right)={p}_{\mathrm{\theta}}\left({x}_T\right){\prod}_{t=1}^T{p}_{\mathrm{\theta}}\left({x}_{t-1}|{x}_t\right) \end{equation*}

These equations have two main differences with the forward diffusion parametrization: (i) the time trajectory is reversed and (ii) the Gaussian distribution parameters must be learned via a parametrized model.

The diffusion model loss function is a negative log-likelihood (NLL) loss that measures the discrepancy between the true data distribution and the learned distribution. Minimizing the NLL given the model parameter is intractable, but it can become tractable via variational inference techniques. Similar to maximizing the ELBO as discussed in the context of VAEs, the evidence lower bound formulation for training diffusion models after applying Bayes rule and simplifying is a tractable loss function shown in equation 10.


(10) \begin{align*} \mathrm{L}&={\mathrm{D}}_{KL}\left(\mathrm{q}\left({\mathrm{x}}_T\;|\;{\mathrm{x}}_0\right)\;\Big\Vert\;\mathrm{p}\left({\mathrm{x}}_T\right)\right)\nonumber \\&+{\sum}_{\mathrm{t}=2}^T{D}_{KL}\left(q\left({x}_{t-1}|\;{x}_t,{x}_0\right)\;\Big\Vert\;{p}_{\theta}\left({x}_{t-1}\;|\;{x}_t\right)\right)\nonumber \\&-\log{p}_{\theta}\left({x}_0|\;{x}_1\right) \end{align*}

In denoising diffusion probabilistic models (DDPM), the authors explored and reformulated the loss function above where the variance was held constant, and the neural network was designed to predict the noise only at each time step. This results in a simple and easily implementable loss function represented in equation 11: the mean squared error between the actually added noise in the forward process and predicted noise by the model.


(11) \begin{equation*} \mathrm{L}={\mathrm{E}}_{t,{x}_0,\epsilon}\left[{\left|\left|\mathrm{\varepsilon} -{\mathrm{\varepsilon}_{\theta}}{({x}_t,t)}\right|\right|}^2\right]\end{equation*}

Diffusion has a more intricate path in model development and refinement compared to the other mentioned generative models. The idea of using a diffusion process in deep unsupervised learning was proposed in 2015 by Sohl-Dickstein *et al.*[35] in which the data distribution was destroyed gradually via an ‘iterative’ forward process and Markov chain method. The authors argued that the reverse diffusion process (restoring data distribution from known distribution (e.g. normal distribution)) yields a tractable generative model when applied with a sufficient number of steps. The reasoning behind this was that small perturbations in data are more tractable for prediction than one-time distribution prediction. In 2020, Ho *et al.*[104] introduced a series of novel enhancements to this technique, leading to high-quality image synthesis through denoizing diffusion probabilistic models (DDPMs). In their research, the authors employed a linear noise scheduler and innovatively chose to predict the image noise during each iteration in the backward process. Building upon these advancements, the model’s performance was further elevated by incorporating $\mathrm{\beta}$ as a learned parameter in the normal distribution instead of a fixed number. Also, introducing non-linear noise-schedulers (i.e. cosine scheduler) resulted in effective preservation of the data distribution in early nosing steps throughout the forward diffusion process [106, 107].

One main breakthrough in diffusion generative model development was made by Song *et al.* [108] by incorporating a stochastic differential equation (SDE) framework. The ‘score function’ in their methodology refers to the gradient of the log probability density. In the forward process, the data distribution gets perturbed in continuous space (in contrast to earlier diffusion models with finite noising steps) via the suggested SDE formulation which does not have trainable parameters. Reverse SDE can be solved analytically with methods like Euler-Maruyama after handling the score function term [109]. The authors addressed this by modeling the score function using a neural network, which then can be plugged into the reverse SDE formula. Equations 12 and 13 show the main forward and reverse formulation used in an SDE process.


(12) \begin{equation*} dx=f\left(x,t\right) dt+g(t) dw \end{equation*}

(13) \begin{equation*} dx=f\left(x,t\right){-} \left[{g}^2(t){\nabla}_x\log{p}_t(x)\right] dt+g(t)d\overline{w} \end{equation*}

The inclusion of SDEs in score-based generative models led to enhanced flexibility, particularly by eliminating the constant prior in favor of utilizing the density gradient. This method provided a controlled generation process and exact likelihood calculations. Although this proposed method enabled efficient and high-quality sampling, the authors noted a slower sampling compared to GANs over their tested dataset.

Diffusion models have been adopted into protein engineering applications recently, and they have shown incredible performance in generating novel protein structures and sequences. In this complex landscape, diffusion models offer distinct advantages among generative models: diversity, fine-grained control in generation, stability in training, a more favorable platform for conditioning, and high compatibility for sequence and structure co-design [110–113]. While they are generally more computationally intensive than other generative models, the probabilistic nature of diffusion models allows for the generation of diverse protein conformations from initial noise distribution. This inherent uncertainty is particularly beneficial and offers a more realistic modeling approach since proteins are dynamic and adopt multiple conformations. Given these unique features in their architecture and training procedure, diffusion models are potentially an invaluable tool for navigating the intricate energy landscape that proteins operate within.

### Protein engineering highlights of diffusion models

Explore Table 6 for an array of recently developed diffusion model applications in protein engineering, extended with two analytical case studies.

**Table 6:** Summary of highlighted applications of diffusion models for protein engineering

Protein Engineering Task	Advancements	Model Type	Training Data Source(s)	Year	Ref.
Protein sequence inpainting and generation	Demonstrated the use of equivariant denoising using the invariant point attention technique to jointly model protein sequence and structures	Transformer-Based Diffusion Model	PDB	May 2022	[115]
Joint Sequence-Structure Generation	Modeled structure and sequence of full protein complexes in a computationally efficient manner	Graph Neural Network-Based Diffusion Model	PDB, UniProt	Dec. 2022	[111]^a^
Protein Backbone Joint Sequence-Structure model development.	Demonstrated the feasibility of developing generative diffusion models through a comparison of sequence-only, structure-only, and join sequence-structure models.	CARP	PDB	May 2023	[116]
Joint Sequence-Structure Generation	Developed a model capable of generating both novel sequences and novel protein backbones via RoseTTAFold	DDPM	INDI, SCOP	May 2023	[112]^a^
De Novo Protein Sequence Generation with desired structural features.	Generated novel protein sequences with desired secondary-structure features using an attention-based diffusion model.	Attention-Based Diffusion Model	[117]	July 2023	[113]
Antibody joint Sequence-structure Modeling	Improved joint protein sequence and structure generation using both domain knowledge and physics-based constraints.	SE(3)-based Diffusion Model	pOAS Database, HER2 Binder data set [118, 119]	July 2023	[120]

^a^See discussion below for a more detailed case study.

#### ProteinGenerator enables the joint generation of protein sequence and structure

The authors implemented DDPM with coordinated guidance on sequence and structure resulting in improved generation (github.com/RosettaCommons/protein_generator) [112]. They leveraged RoseTTAFold’s [114] capability to simultaneously generate protein sequences and structures. Drawing inspiration from RoseTTAFold Joint Inpainting, they adopted this ability for the diffusive creation of consistent sequence-structure pairs. Fine-tuning to retrieve noised native protein sequences and simultaneously ensuring the accuracy of structure prediction enabled guidance from both sequential and structural domains. In the unconditional generation, ProteinGenerator was able to generate pairs of sequence structures close to the native proteins. Note that various structural properties and amino acid frequencies were obtained by sampling from different noise distributions. The model architecture also enabled high versatility and as a result compatibility with different conditioning and classifier-guidance methods. In conditioning, additional constraints were added to the generation process. For instance, the model was conditioned for generating high amino acid frequencies (e.g. cystine for forming disulfide bonds to increase stability, histidine for pH sensitivity) while satisfying the corresponding structure folding. In another example, DeepGOPlus Gene Ontology (GO) classifier was used to guide the generation process. The classifier provides scores or gradients that can be used to influence the outputs of the main model and as a result, generate functionally rich sequences.

#### Chroma enables the generation of novel protein complexes via its join sequence-structure model

Another highly successful implementation of the diffusion-based framework was shown in Chroma which enabled jointly modeling the sequence and structure of full protein complexes (https://github.com/lucidrains/chroma-pytorch) [111]. The authors introduced sophisticated computational techniques and conditional sampling to adeptly manage computational challenges while crafting proteins with specific attributes. Rooted in diffusion modeling and graph neural networks, this versatile generative model excels in refining noisy structures while preserving the intricate 3D details inherent in protein configurations. This model facilitates programmable protein design as it can condition proteins on different shapes, symmetry, textual prompts, and various properties. Remarkably, Chroma’s capability to generate protein complexes holds significant value as most of the protein functions such as binding occur through protein interactions. Furthermore, the authors indicated that a large protein (e.g. with > 3000 residues) can be generated within minutes via an appropriate GPU (e.g. NVIDIA V100).

## DISCUSSION

Generative models—such as VAEs, autoregressive, GANs, and diffusion models—have shown significant promise in the protein engineering domain to generate novel and functional sequences. This ongoing research has mitigated long-standing challenges in designing proteins with improved properties, generating interfaces for protein–protein interactions, establishing rules for high-fitness protein variants and capturing phylogenetic relationships between proteins. These models aim to learn the underlying data distribution and generate novel instances via sampling from the learned distribution. Distinct model structures are employed to learn the given data distribution by directly modeling or approximating the probability density function. VAEs are probabilistic generative models that approximate the explicit density function via variational inference. Upon learning the underlying distribution of the given dataset, the VAE can generate novel samples similar to input data. VAEs have been used in various protein engineering tasks including improving fitness (e.g. thermal stability, solubility, bioluminescence and binding) and capturing phylogenetic relationships via learned latent space relationships. Autoregressive models calculate the explicit density function where each token is conditioned on the previous tokens. Autoregressive models have also led to successful outcomes in generating sequences with improved fitness, paratope prediction, and protein localization. Unlike VAEs and autoregressive models that use restricted neural networks in approaching the intractable normalizing constant, GANs model the generation process only. As a result, they are not used for likelihood estimation, yet they have superior potential in generating high-quality instances. Two networks (generator and discriminator) are used in GANs that sample from the density function without calculating or estimating the function itself (i.e. implicit density estimation). GANs provide promising results in diverse tasks such as gene ontology correlation, binding affinity, phylogeny prediction, antimicrobial peptide generation and developing rules for antibody solubility and thermal stability. Diffusions are a more recent class of generative models adopted from thermodynamics equilibrium. The idea is if the noise in the data happens gradually, it can be reversed. Therefore, data distribution can be approximated from pure noise in the reverse diffusion process.

While each of these generative models has obtained promising outcomes in terms of protein design applications, they differ in their training process, output quality and generated output diversity. In general, given their efficient architecture, VAEs are potentially easier to train, yet they might lead to lower quality outputs (e.g. blurry images for image generation) compared to other generative models [121]. Note that recent architecture developments have tried to overcome common issues in VAEs (e.g. posterior collapse and reconstruction-regularization trade off), yet these solutions may require more computational resources. For instance, beta-VAE [85], hierarchical VAE [122, 123] and VQ-VAE [124] are distinct types of VAE models to address common issues in traditional VAEs. Beta-VAE adds a hyperparameter in the loss function to obtain more disentangled representations. Hierarchical VAE aims to preclude posterior over-regularization by incorporating hierarchical priors in the model. Finally, VQ-VAE has been shown to generate high-quality data and prevent posterior collapse by learning discrete representations and autoregressive prior (versus continuous learned representations and static prior in original traditional VAE). Similarly, there are improved variants for GANs and autoregressive models to boost generated data attributes and resolve model restrictions. Examples of autoregressive developments include GPT-3 [59], Reformer [125] and Big Bird [126] which use more parameters in training, reversible sequence-to-sequence architecture, and sparse attention mechanism, respectively. For GANS, improved variants include CycleGAN [127], LsGAN [128] and VEEGAN [129] for training without paired data, resolving vanishing gradient issues in training and reducing mode collapse to increase generated data diversity, respectively. Although diffusion models have been developed recently, their architecture is rapidly evolving. For example, subspace diffusion has shown improved sampling quality and reduced computational cost via restricting diffusion by its projection to subspaces [130]. Denoising diffusion policy optimization (DDPO) is another architecture development in diffusions which solved the denoising process as a multi-step decision-making problem [131]. The mentioned architectures are a few variants among a pool of architectures and their performance depends on the specific application and data attributes (e.g. number of samples in training, data complexity and input data length).

Despite the newfound opportunities provided by generative models in this realm, the remaining challenges in generative sequence modeling include validating the generated sequences, navigating the rugged landscape in pursuit of sequences with desired features, de-novo binder design applications, effectively infusing biological priors into models and strategically combining distinct generative models to enhance sampling quality and diversity. In many cases, wet-lab experiments are required to assess the quality of the generated sequence in terms of basic required properties (e.g. stability and expression) to more design-based properties (e.g. affinity and specificity). This by itself has hindered model optimization as there is no immediate and definitive feedback for the quality of generated sequences (versus rapidly assessing the visual quality of a general image-based data generated from these models). With that being said, there are computational tools to aid in filtering the generated sequences and increasing the success rate in experimental characterization. For example, Alphafold2 for structural prediction [132], discriminative models to assign probabilities to sequences based on their fitness [133], and self-supervised models for few and zero-shot predictions [134] are among the extremely beneficial tools for analyzing the generated sequences *in silico*.

In this paper, we provided an overview of the architecture and underlying assumptions of four commonly used generative models (VAEs, Autoregressive models, GANs and diffusion models). By analyzing the strengths and limitations of each model, we hope that researchers are better equipped to make informed decisions when selecting the appropriate model for specific data and objectives. We also elaborated on specific protein engineering applications for each of these models, highlighting their potential to generate novel protein sequences with improved properties. With the exponential growth of biological and protein sequence datasets, increasing efficiency of generative models, and improved methods for generating and validating *de novo* sequences, we envision a promising future for the development of effective protein design and engineering applications.

**Key Points**

- To address the gap between the growing number of machine learning (ML) models and their application to protein engineering tasks, we have reviewed recent protein engineering applications of generative ML models.
- The architecture and mathematical background of three generative models (diffusion models, generative adversarial neural networks and variational autoencoders) are described in depth with a focus on applications towards protein design (e.g. to predict protein properties and to generate protein design rules and sequences).
- The architecture and application of language machine learning models (namely, recurrent neural networks, autoregressive and transformers) are also described, particularly in the context of treating protein design tasks on amino acid sequences similarly to human language tasks on strings of text.
- Incorporating transfer learning and embeddings can improve the efficiency and generalizability of ML modeling tasks.

## Data Availability

Not applicable.

## Notes

**Mehrsa Mardikoraem** is a PhD candidate in Michigan State University (MSU)‘s Department of Chemical Engineering and Materials Science where her thesis research focuses on leveraging ML tools for rationally-guided library design of therapeutic and diagnostic proteins.

**Zirui Wang** is a Data Engineer at Regeneron Pharmaceuticals, Inc. Having received his B.S. in Chemical Engineering from MSU, he is currently pursuing a M.S. in Computer Science from Syracuse University. As a member of the Woldring Lab, he developed a pipeline for ML-assisted *in silico* screening of phytochemical candidates against SARS-CoV-2.

**Nathaniel Pascual**, recently graduated with a B.S. in Chemical Engineering from MSU, is a research assistant in the Woldring Lab where he investigates high-throughput methods to characterize membrane proteins and integrating single-cell sequencing for antibody design.

**Daniel Woldring** is an assistant professor in MSU’s Department of Chemical Engineering and Materials Science and a member of MSU’s Institute for Quantitative Health Sciences and Engineering. His research combines high-throughput wet lab methods with ML-driven computational tools to design proteins.

## References

[1] Webster JM , Zhang R, Gambhir SS, et al. Engineered two-helix small proteins for molecular recognition. Chem Bio Chem 2009;10:1293–6. 10.1002/cbic.200900062 19422008

[2] Eke CS , Jammeh E, Li X, et al. Early detection of Alzheimer’s disease with blood plasma proteins using support vector machines. IEEE J Biomed Health Inform 2021;25:218–26. 32340968 10.1109/JBHI.2020.2984355

[3] Luan Y , Yao Y. The clinical significance and potential role of C-reactive protein in chronic inflammatory and neurodegenerative diseases. Front Immunol 2018;9:1302. 29951057 10.3389/fimmu.2018.01302 PMC6008573

[4] Bam R , Lown PS, Stern LA, et al. Efficacy of Affibody-based ultrasound molecular imaging of vascular B7-H3 for breast cancer detection. Clin Cancer Res 2020;26:2140–50. 31924738 10.1158/1078-0432.CCR-19-1655 PMC7196517

[5] Małecki J , Muszyński S, Sołowiej BG. Proteins in food systems—bionanomaterials, conventional and unconventional sources, functional properties, and development opportunities. Polymers 2021;13:2506. 34372109 10.3390/polym13152506 PMC8347159

[6] Janssen DB , Schanstra JP. Engineering proteins for environmental applications. Curr Opin Biotechnol 1994;5:253–9. 7765007 10.1016/0958-1669(94)90026-4

[7] Kuroda K , Ueda M. Molecular Design of the Microbial Cell Surface toward the recovery of metal ions. Curr Opin Biotechnol 2011;22:427–33. 21247751 10.1016/j.copbio.2010.12.006

[8] Prakash D , Gabani P, Chandel AK, et al. Bioremediation: a genuine technology to remediate radionuclides from the environment. J Microbial Biotechnol 2013;6:349–60. 10.1111/1751-7915.12059 PMC3917470 23617701

[9] Jez JM . Toward protein engineering for phytoremediation: possibilities and challenges. Int J Phytoremediation 2011;13:77–89. 22046752 10.1080/15226514.2011.568537

[10] Jia X , Li Y, Xu T, Wu K. Display of lead-binding proteins on *Escherichia coli* surface for lead bioremediation. Biotechnol Bioeng 2020;117:3820–34. 32740905 10.1002/bit.27525

[11] Diem MD , Hyun L, Yi F, et al. Selection of high-affinity Centyrin FN3 domains from a simple library diversified at a combination of strand and loop positions. Protein Eng Des Sel 2014;27:419–29. 24786107 10.1093/protein/gzu016

[12] Golinski AW , Mischler KM, Laxminarayan S, et al. High-throughput developability assays enable library-scale identification of producible protein scaffold variants. Proc Natl Acad Sci 2021;118:e2026658118. 34078670 10.1073/pnas.2026658118 PMC8201827

[13] Zacharias M . Protein–protein docking with a reduced protein model accounting for side-chain flexibility. Protein Sci 2003;12:1271–82. 12761398 10.1110/ps.0239303 PMC2323887

[14] Merkl R , Sterner R. Reconstruction of ancestral enzymes. Perspect Sci 2016;9:17–23.

[15] Vaswani A , Shazeer N, Parmar N, et al. Attention Is All You Need, 2017. arXiv preprint, arXiv:1706.03762.

[16] Ghojogh B , Ghodsi A. Attention Mechanism, Transformers, BERT, and GPT: Tutorial and Survey. Charlottesville, VA: Open Science Framework, 2020.

[17] LeCun Y , Bengio Y, Hinton G. Deep learning. Nature 2015;521:436–44. 26017442 10.1038/nature14539

[18] Ofer D , Brandes N, Linial M. The language of proteins: NLP, machine learning & protein sequences. Comput Struct Biotechnol J 2021;19:1750–8. 33897979 10.1016/j.csbj.2021.03.022 PMC8050421

[19] Wang K , Zhou R, Li Y, Li M. DeepDTAF: a deep learning method to predict protein–ligand binding affinity. Brief Bioinform 2021;22:bbab072. 33834190 10.1093/bib/bbab072

[20] Li G , Rabe KS, Nielsen J, Engqvist MKM. Machine learning applied to predicting microorganism growth temperatures and enzyme catalytic optima. ACS Synth Biol 2019;8:1411–20. 31117361 10.1021/acssynbio.9b00099

[21] Khurana S , Rawi R, Kunji K, et al. DeepSol: a deep learning framework for sequence-based protein solubility prediction. Bioinformatics 2018;34:2605–13. 29554211 10.1093/bioinformatics/bty166 PMC6355112

[22] Hashemifar S , Neyshabur B, Khan AA, Xu J. Predicting protein–protein interactions through sequence-based deep learning. Bioinformatics 2018;34:i802–10. 30423091 10.1093/bioinformatics/bty573 PMC6129267

[23] Wang L , Wang H-F, Liu S-R, et al. Predicting protein-protein interactions from matrix-based protein sequence using convolution neural network and feature-selective rotation Forest. Sci Rep 2019;9:9848. 31285519 10.1038/s41598-019-46369-4 PMC6614364

[24] Ferruz N , Schmidt S, Höcker B. A deep unsupervised language model for protein design. 2022;2022.03.09.483666. 10.1038/s41467-022-32007-7 PMC9329459 35896542

[25] Rao R , Bhattacharya N, Thomas N, et al. Evaluating protein transfer learning with TAPE. Adv Neural Inf Process Syst 2019;32:9689–701. 33390682 PMC7774645

[26] Alley EC , Khimulya G, Biswas S, et al. Unified rational protein engineering with sequence-based deep representation learning. Nat Methods 2019;16:1315–22. 31636460 10.1038/s41592-019-0598-1 PMC7067682

[27] Rives A , Meier J, Sercu T, et al. Biological structure and function emerge from scaling unsupervised learning to 250 million protein sequences. Proc Natl Acad Sci 2021;118:e2016239118. 33876751 10.1073/pnas.2016239118 PMC8053943

[28] Elnaggar A , Heinzinger M, Dallago C, et al. ProtTrans: toward understanding the language of life through self-supervised learning. IEEE Trans Pattern Anal Mach Intell 2022;44:7112–27. 34232869 10.1109/TPAMI.2021.3095381

[29] Costello Z , Martin HG. How to Hallucinate Functional Proteins, 2019. arXiv, arXiv:1903.00458v1.

[30] Ferruz N , Schmidt S, Höcker B. ProtGPT2 is a deep unsupervised language model for protein design. Nat Commun 2022;13:4348. 35896542 10.1038/s41467-022-32007-7 PMC9329459

[31] Repecka D , Jauniskis V, Karpus L, et al. Expanding functional protein sequence spaces using generative adversarial networks. Nat Mach Intell 2021;3:324–33.

[32] Watson JL , Juergens D, Bennett NR, et al. De novo design of protein structure and function with RF diffusion. Nature 2023;620:1089–100. 37433327 10.1038/s41586-023-06415-8 PMC10468394

[33] Kingma DP , Welling M. Auto-Encoding Variational Bayes, 2022. arXiv, arXiv:1312.6114v11.

[34] Goodfellow IJ , Pouget-Abadie J, Mirza M, et al. Generative Adversarial Networks, 2014. arXiv; https://arxiv.org/abs/1406.2661.

[35] Sohl-Dickstein, J.; Weiss, E.; Maheswaranathan, N.; Ganguli, S. Deep Unsupervised Learning Using Nonequilibrium Thermodynamics. In: Francis Bach, David Blei (eds) Proceedings of the Proceedings of the 32nd International Conference on Machine Learning. France: PMLR, June 1 2015; pp. 2256–65.

[36] Sherstinsky A . Fundamentals of recurrent neural network (RNN) and long short-term memory (LSTM) network. Phys Nonlinear Phenom 2020;404:132306.

[37] Schuster M , Paliwal KK. Bidirectional recurrent neural networks. IEEE Trans Signal Process 1997;45:2673–81.

[38] Hochreiter S , Schmidhuber J. Long short-term memory. Neural Comput 1997;9:1735–80. 9377276 10.1162/neco.1997.9.8.1735

[39] Chung J , Gulcehre C, Cho K, Bengio Y. Empirical Evaluation of Gated Recurrent Neural Networks on Sequence Modeling, 2014. arXiv; arXiv:1412.3555.

[40] Müller AT , Hiss JA, Schneider G. Recurrent neural network model for constructive peptide design. J Chem Inf Model 2018;58:472–9. 29355319 10.1021/acs.jcim.7b00414

[41] Saka K , Kakuzaki T, Metsugi S, et al. Antibody design using LSTM based deep generative model from phage display library for affinity maturation. Sci Rep 2021;11:5852. 33712669 10.1038/s41598-021-85274-7 PMC7955064

[42] Sabban S , Markovsky M. RamaNet: computational de novo helical protein backbone design using a long short-term memory generative neural network. F1000 Research Full 2020;9:671552.

[43] Zhang B , Li J, Lü Q. Prediction of 8-state protein secondary structures by a novel deep learning architecture. BMC Bioinformatics 2018;19:293. 30075707 10.1186/s12859-018-2280-5 PMC6090794

[44] Lin C-C , Jaech A, Li X, et al. In: Kristina Toutanova, Anna Rumshisky, Luke Zettlemoyer, Dilek Hakkani-Tur, Iz Beltagy, Steven Bethard, Ryan Cotterell, Tanmoy Chakraborty, Yichao Zhou (eds) Limitations of Autoregressive Models and Their Alternatives. Kerrville, TX: Association for Computational Linguistics, 2021.

[45] Trinquier J , Uguzzoni G, Pagnani A, et al. Efficient generative Modeling of protein sequences using simple autoregressive models. Nat Commun 2021;12:5800. 34608136 10.1038/s41467-021-25756-4 PMC8490405

[46] Shin J-E , Riesselman AJ, Kollasch AW, et al. Protein design and variant prediction using autoregressive generative models. Nat Commun 2021;12:2403. 33893299 10.1038/s41467-021-22732-w PMC8065141

[47] Zhang, P.; Zheng, S.; Chen, J.; Zhou, Y.; Yang, Y. DeepANIS: Predicting Antibody Paratope from Concatenated CDR Sequences by Integrating Bidirectional Long-Short-Term Memory and Transformer Neural Networks. In: Yufei Huang, Lukasz Kurgan, Feng Luo, Xiaohua Hu, Yidong Chen, Edward Dougherty, Andrzej Kloczkowski, Yaohang Li (eds) Proceedings of the 2021 IEEE International Conference on Bioinformatics and Biomedicine (BIBM). New York, NY: IEEE, 2021; pp. 118–24.

[48] Liu X . Deep Recurrent Neural Network for Protein Function Prediction from Sequence, 2017. arXiv; arXiv:1701.08318.

[49] Panda B , Majhi B. A novel improved prediction of protein structural class using deep recurrent neural network. Evol Intell 2021;14:253–60.

[50] Russ WP , Figliuzzi M, Stocker C, et al. An evolution-based model for designing Chorismate mutase enzymes. Science 2020;369:440–5. 32703877 10.1126/science.aba3304

[51] Grechishnikova D . Transformer neural network for protein-specific de novo drug generation as a machine translation problem. Sci Rep 2021;11:321. 33432013 10.1038/s41598-020-79682-4 PMC7801439

[52] Wu Z , Yang KK, Liszka MJ, et al. Signal peptides generated by attention-based neural networks. ACS Synth Biol 2020;9:2154–61. 32649182 10.1021/acssynbio.0c00219

[53] Ieremie I , Ewing RM, Niranjan M. TransformerGO: predicting protein–protein interactions by modelling the attention between sets of gene ontology terms. Bioinformatics 2022;38:2269–77. 35176146 10.1093/bioinformatics/btac104 PMC9363134

[54] Chen C , Wu T, Guo Z, Cheng J. Combination of deep neural network with attention mechanism enhances the explainability of protein contact prediction. Proteins Struct Funct Bioinforma 2021;89:697–707. 10.1002/prot.26052 PMC8089057 33538038

[55] O’Shea K , Nash R. An Introduction to Convolutional Neural Networks, 2015. arXiv; arXiv:1511.08458.

[56] Zhao Q , Zhao H, Zheng K, Wang J. HyperAttentionDTI: improving drug–protein interaction prediction by sequence-based deep learning with attention mechanism. Bioinformatics 2022;38:655–62. 34664614 10.1093/bioinformatics/btab715

[57] Devlin J , Chang M-W, Lee K, Toutanova K. BERT: Pre-Training of Deep Bidirectional Transformers for Language Understanding. In: Jill Burstein, Christy Doran, Thamar Solorio (eds). Minneapolis, Minnesota: Association for Computational Linguistics, 2019.

[58] Radford A , Narasimhan K, Salimans T, Sutskever I. Improving language understanding by generative pre-training.

[59] Brown TB , Mann B, Ryder N, et al. Language models are few-shot learners. In: Larochelle H, Ranzato M, Hadsell R, Balcan MF, Lin H (eds) Advances in Neural Information Processing Systems 33 (NeurIPS 2020). 2020;33:1877–1901.

[60] Tsimpoukelli, M.; Menick, J.L.; Cabi, S. et al. . Multimodal Few-Shot Learning with Frozen Language Models. In: Ranzato M, Beygelzimer A, Dauphin Y, Liang PS, Wortman Vaughan J (eds) Proceedings of the Advances in Neural Information Processing Systems; Curran Associates, Inc.. Virtual: Neural Information Processing Systems, 2021; Vol. 34, pp. 200–12.

[61] Lewis M , Liu Y, Goyal N, et al. BART: Denoising sequence-to-sequence pre-training for natural language generation. Transl Comprehen 2019;58:7871–80.

[62] Choromanski K , Likhosherstov V, Dohan D, et al. Masked Language Modeling for Proteins via Linearly Scalable Long-Context Transformers, 2020. arXiv; arXiv:2006.03555.

[63] Min S , Park S, Kim S, et al. Pre-training of deep bidirectional protein sequence representations with structural information. IEEE Access 2021;9:123912–26.

[64] Cao R , Freitas C, Chan L, et al. ProLanGO: protein function prediction using neural machine translation based on a recurrent neural network. Molecules 2017;22:1732. 29039790 10.3390/molecules22101732 PMC6151571

[65] Hu S , Ma R, Wang H. An improved deep learning method for predicting DNA-binding proteins based on contextual features in amino acid sequences. PloS One 2019;14:e0225317. 31725778 10.1371/journal.pone.0225317 PMC6855455

[66] Johnson SR , Monaco S, Massie K, Syed Z. Generating novel protein sequences using Gibbs sampling of masked language models. 2021;2021.01.26.428322. https://www.biorxiv.org/content/10.1101/2021.01.26.428322v1.

[67] Notin, P.; Dias, M.; Frazer, J. et al.. Tranception: Protein Fitness Prediction with Autoregressive Transformers and Inference-Time Retrieval. In: Kamalika Chaudhuri, Stefanie Jegelka, Le Song, Csaba Szepesvari, Gang Niu, Sivan Sabato (eds) Proceedings of the Proceedings of the 39th International Conference on Machine Learning. Baltimore, MDE: PMLR, June 28 2022; pp. 16990–7017.

[68] Castro E , Godavarthi A, Rubinfien J, et al. Transformer-based protein generation with regularized latent space optimization. Nat. Mach. Intell. 2022;4:840–51.

[69] Pan SJ , Yang Q. A survey on transfer learning. IEEE Trans Knowl Data Eng 2010;22:1345–59.

[70] UniProt: A Hub for Protein Information . Nucleic Acids Research. Oxford, UK: Oxford Academic Available online: https://academic.oup.com/nar/article/43/D1/D204/2439939 (accessed on 28 March 2023). 10.1093/nar/gku989 PMC4384041 25348405

[71] Suzek BE , Huang H, McGarvey P, et al. UniRef: comprehensive and non-redundant UniProt reference clusters. Bioinformatics 2007;23:1282–8. 17379688 10.1093/bioinformatics/btm098

[72] Katz K , Shutov O, Lapoint R, et al. The sequence read archive: a decade more of explosive growth. Nucleic Acids Res 2022;50:D387–90. 34850094 10.1093/nar/gkab1053 PMC8728234

[73] Biswas S , Khimulya G, Alley EC, et al. Low-N protein engineering with data-efficient deep learning. Nat Methods 2021;18:389–96. 33828272 10.1038/s41592-021-01100-y

[74] Lin Z , Akin H, Rao R, et al. Evolutionary-scale prediction of atomic-level protein structure with a language model. Science 2023;379:1123–30. 36927031 10.1126/science.ade2574

[75] Heinzinger M , Elnaggar A, Wang Y, et al. Modeling aspects of the language of life through transfer-learning protein sequences. BMC Bioinformatics 2019;20:723. 31847804 10.1186/s12859-019-3220-8 PMC6918593

[76] Nambiar A , Liu S, Hopkins M, et al. Transforming the language of life: transformer neural networks for protein prediction tasks. Journal of Computational Biology 2020;30:95–111. 10.1089/cmb.2022.0132 35950958

[77] Vig J , Madani A, Varshney LR, et al. BERTology meets biology: interpreting attention in protein language models. 2021. arXiv; arXiv:2006.15222.

[78] He L , Zhang S, Wu L, et al. Pre-Training Co-Evolutionary Protein Representation via A Pairwise Masked Language Model, 2021. arXiv; arXiv:2110.15527.

[79] Behjati A , Zare-Mirakabad F, Arab SS, Nowzari-Dalini A. Protein sequence profile prediction using ProtAlbert transformer. Computational Biology and Chemistry 2021;99. 2021.09.23.461475. 10.1016/j.compbiolchem.2022.107717 35802991

[80] Mardikoraem M , Woldring D. Protein fitness prediction is impacted by the interplay of language models, ensemble learning, and sampling methods. Pharmaceutics 2023;15(5). 2023.02.09.527362. 10.3390/pharmaceutics15051337 PMC10224321 37242577

[81] Shanehsazzadeh A , Belanger D, Dohan D. Is Transfer Learning Necessary for Protein Landscape Prediction? 2020. arXiv; arXiv:2011.03443.

[82] Wittmann BJ , Yue Y, Arnold FH. Informed training set design enables efficient machine learning-assisted directed protein evolution. Cell Syst. 2021;12:1026–1045.e7. 34416172 10.1016/j.cels.2021.07.008

[83] Sinai S , Kelsic E, Church GM, Nowak MA. Variational Auto-Encoding of Protein Sequences, 2018. arXiv; arXiv:1712.03346.

[84] Blei DM , Kucukelbir A, McAuliffe JD. Variational inference: a review for statisticians. J Am Stat Assoc 2017;112:859–77.

[85] Higgins I , Matthey L, Pal A, et al. Beta-VAE: learning basic visual concepts with a constrained variational framework. Conference Paper for International Conference on Learning Representations (ICLR) 2022;5.

[86] Razavi A , Vinyals O. Preventing Posterior Collapse with δ-VAES, 2019. arXiv; arXiv:1901.03416.

[87] Davidsen K , Olson BJ, DeWitt WS, III, et al. IV deep generative models for T cell receptor protein sequences. Elife 2019;8:e46935. 31487240 10.7554/eLife.46935 PMC6728137

[88] Greener JG , Moffat L, Jones DT. Design of metalloproteins and novel protein folds using variational autoencoders. Sci Rep 2018;8:16189. 30385875 10.1038/s41598-018-34533-1 PMC6212568

[89] Albu, A.-I.; Czibula, G. Analysing protein dynamics using machine learning based generative models. In Proceedings of the 2020 IEEE 14th International Symposium on Applied Computational Intelligence and Informatics (SACI); 2020;14:000135–40.

[90] Linder J , Bogard N, Rosenberg AB, Seelig G. A generative neural network for maximizing fitness and diversity of synthetic DNA and protein sequences. Cell Syst 2020;11:49–62.e16. 32711843 10.1016/j.cels.2020.05.007 PMC8694568

[91] Hawkins-Hooker A , Depardieu F, Baur S, et al. Generating functional protein variants with variational autoencoders. PLoS Comput Biol 2021;17:e1008736. 33635868 10.1371/journal.pcbi.1008736 PMC7946179

[92] McGee F , Hauri S, Novinger Q, et al. The generative capacity of probabilistic protein sequence models. Nat Commun 2021;12:6302. 34728624 10.1038/s41467-021-26529-9 PMC8563988

[93] Moreta LS , Rønning O, Al-Sibahi AS. Ancestral Protein Sequence Reconstruction Using a Tree-Structured Ornstein-Uhlenbeck Variational Autoencoder. Virtual: International Conference on Learning Representations, 2022. PMC12908695 41704296

[94] Arjovsky M , Chintala S, Bottou L. Wasserstein GAN, 2017. arXiv; arXiv:1701.07875.

[95] Wan C , Jones DT. Protein function prediction is improved by creating synthetic feature samples with generative adversarial networks. Nat. Mach. Intell. 2020;2:540–50.

[96] Gupta A , Zou J. Feedback GAN for DNA optimizes protein functions. Nat Mach Intell 2019;1:105–11.

[97] Davis MI , Hunt JP, Herrgard S, et al. Comprehensive analysis of kinase inhibitor selectivity. Nat Biotechnol 2011;29:1046–51. 22037378 10.1038/nbt.1990

[98] Zhao L , Wang J, Pang L, et al. GANsDTA: predicting drug-target binding affinity using GANs. Front Genet 2020;10:1243. 10.3389/fgene.2019.01243 PMC6962343 31993067

[99] Amimeur T , Shaver JM, Ketchem RR, et al. Designing feature-controlled humanoid antibody discovery libraries using generative adversarial networks. 2020; 2020.04.12.024844. 10.1101/2020.04.12.024844.

[100] Berman DS , Howser C, Mehoke T, Evans JD. MutaGAN: A Seq2seq GAN Framework to Predict Mutations of Evolving Protein Populations. Oxford, UK: Virus Evolution, 2020. 10.1093/ve/vead022 PMC10104372 37066021

[101] Seyyedsalehi SF , Soleymani M, Rabiee HR, Mofrad MRK. PFP-WGAN: protein function prediction by discovering gene ontology term correlations with generative adversarial networks. PloS One 2021;16:e0244430. 33630862 10.1371/journal.pone.0244430 PMC7906332

[102] Ramesh A , Dhariwal P, Nichol A, et al. Hierarchical Text-Conditional Image Generation with CLIP Latents, 2022. arXiv; arXiv:2204.06125.

[103] Ramesh, A.; Pavlov, M.; Goh, G. et al. Zero-Shot Text-to-Image Generation. In: Marina Meila, Tong Zhang (eds) Proceedings of the Proceedings of the 38th International Conference on Machine Learning. Virtual: PMLR, July 1 2021; pp. 8821–8831.

[104] Ho J , Jain A, Abbeel P. Denoising Diffusion Probabilistic Models, 2020. arXiv; arXiv:2006.11239.

[105] Weng W , Zhu X. INet: convolutional networks for biomedical image segmentation. IEEE Access 2021;9:16591–603.

[106] Nichol A , Dhariwal P. Improved Denoising Diffusion Probabilistic Models, 2021. arXiv; arXiv:2102.09672.

[107] Dhariwal, P.; Nichol, A. Diffusion models beat GANs on image synthesis. In Proceedings of the Advances in Neural Information Processing Systems; Curran Associates, Inc., 2021; Vol. 34, pp. 8780–94.

[108] Song H , Bremer BJ, Hinds EC, et al. Inferring protein sequence-function relationships with large-scale positive-Unlabeled learning. Cell Syst 2021;12:92–101.e8. 33212013 10.1016/j.cels.2020.10.007 PMC7856229

[109] Bayram M , Partal T, Orucova Buyukoz G. Numerical methods for simulation of stochastic differential equations. Adv Differ Equ 2018;2018:17.

[110] Yang L , Zhang Z, Song Y, et al. Diffusion models: a comprehensive survey of methods and applications. 2023. arXiv; arXiv:2209.00796.

[111] Ingraham J , Baranov M, Costello Z, et al. Illuminating protein space with a programmable generative model. bioRxiv 2022;2022(12):01.518682. 10.1038/s41586-023-06728-8 PMC10686827 37968394

[112] Lisanza SL , Gershon JM, Tipps S, et al. Joint generation of protein sequence and structure with RoseTTAFold sequence space diffusion. 2023; 2023.05.08.539766. 10.1101/2023.05.08.539766.

[113] Ni B , Kaplan DL, Buehler MJ. Generative design of de novo proteins based on secondary-structure constraints using an attention-based diffusion model. Chem 2023;9:1828–49. 37614363 10.1016/j.chempr.2023.03.020 PMC10443900

[114] Baek M , DiMaio F, Anishchenko I, et al. Accurate prediction of protein structures and interactions using a three-track neural network. Science 2021;373:871–6. 34282049 10.1126/science.abj8754 PMC7612213

[115] Anand N , Achim T. Protein Structure and Sequence Generation with Equivariant Denoising Diffusion Probabilistic Models, 2022. arXiv; arXiv:2205.15019.

[116] Vinod R , Yang KK, Crawford L. Joint protein sequence-structure co-design via Equivariant diffusion. November 28 2022.

[117] Yu C-H , Chen W, Chiang Y-H, et al. End-to-end deep learning model to predict and design secondary structure content of structural proteins. ACS Biomater Sci Eng 2022;8:1156–65. 35129957 10.1021/acsbiomaterials.1c01343 PMC9347213

[118] Mason DM , Friedensohn S, Weber CR, et al. Optimization of therapeutic antibodies by predicting antigen specificity from antibody sequence via deep learning. Nat Biomed Eng 2021;5:600–12. 33859386 10.1038/s41551-021-00699-9

[119] Olsen TH , Boyles F, Deane CM. Observed antibody space: a diverse database of cleaned, annotated, and translated unpaired and paired antibody sequences. Protein Sci Publ Protein Soc 2022;31: 141–6. 10.1002/pro.4205 PMC8740823 34655133

[120] Martinkus K , Ludwiczak J, Cho K, et al. AbDiffuser: full-atom generation of in-vitro functioning antibodies. 2023. arXiv; arXiv:2308.05027.

[121] Dosovitskiy, A.; Brox, T. Generating images with perceptual similarity metrics based on deep networks. In Proceedings of the Advances in Neural Information Processing Systems; Curran Associates, Inc., 2016; Vol. 29.

[122] Klushyn A , Chen N, Kurle R, et al. Learning Hierarchical Priors in VAEs, 2019. arXiv; arXiv:1905.04982.

[123] Sønderby CK , Raiko T, Maaløe L, et al. Ladder Variational Autoencoders, 2016. arXiv; arXiv:1602.02282.

[124] van den Oord A , Vinyals O, Kavukcuoglu K. Neural Discrete Representation Learning, 2018. arXiv; arXiv:1711.00937.

[125] Kitaev N , Kaiser Ł, Levskaya A. Reformer: the efficient transformer. 2020. arXiv; arXiv:2001.04451.

[126] Zaheer, M.; Guruganesh, G.; Dubey, K.A. et al. Big bird: transformers for longer sequences. In Proceedings of the Advances in Neural Information Processing Systems; Curran Associates, Inc., 2020; Vol. 33, pp. 17283–97.

[127] Zhu J-Y , Park T, Isola P, Efros AA. Unpaired Image-to-Image Translation Using Cycle-Consistent Adversarial Networks, 2020. arXiv; arXiv:1703.10593.

[128] Mao X , Li Q, Xie H, et al. Least Squares Generative Adversarial Networks, 2017. arXiv; arXiv:1611.04076. 10.1109/TPAMI.2018.2872043 30273144

[129] Srivastava A , Valkov L, Russell C, et al. VEEGAN: Reducing Mode Collapse in GANs Using Implicit Variational Learning. In: Guyon I, Von Luxburg U, Bengio S, Wallach H, Fergus R, Vishwanathan S, Garnett R (eds) Proceedings of the Advances in Neural Information Processing Systems, Vol. 30. Long Beach, CA: Curran Associates, Inc., 2017.

[130] Jing B , Corso G, Berlinghieri R, Jaakkola T. Subspace Diffusion Generative Models. In: Avidan S, Brostow G, Cissé M et al. (eds). Computer Vision – ECCV 2022, Vol. 13683. Cham: Lecture Notes in Computer Science; Springer Nature Switzerland, 2022, 274–89 ISBN 978-3-031-20049-6.

[131] Black K , Janner M, Du Y, et al. Training Diffusion Models with Reinforcement Learning, 2023. arXiv; arXiv:2305.13301.

[132] Jumper J , Evans R, Pritzel A, et al. Highly accurate protein structure prediction with AlphaFold. Nature 2021;596:583–9. 34265844 10.1038/s41586-021-03819-2 PMC8371605

[133] Strokach A , Kim PM. Deep generative modeling for protein design. Curr Opin Struct Biol 2022;72:226–36. 34963082 10.1016/j.sbi.2021.11.008

[134] Meier J , Rao R, Verkuil R, et al. Language models enable zero-shot prediction of the effects of mutations on protein function. 2021;34:2021.07.09.450648.

